# PDCD11 Stabilizes C‐MYC Oncoprotein by Hindering C‐MYC‐SKP2 Negative Feedback Loop to Facilitate Progression of p53‐Mutant Breast and Colon Malignancies

**DOI:** 10.1002/advs.202502416

**Published:** 2025-03-07

**Authors:** Li Ding, Wei Ni, Yichao Ma, Lin Xu, Zhiping Zhang, Kai Liao, Jingwen Li, Xinyu Mei, Zhun Wang, Huiqian Ge, Jiajia Li, Dong Tang, Xinyue Zhang

**Affiliations:** ^1^ College of Bioscience and Biotechnology Yangzhou University Yangzhou 225009 China; ^2^ Department of General Surgery Institute of General Surgery Northern Jiangsu People's Hospital Affiliated to Yangzhou University Yangzhou 225001 China

**Keywords:** C‐MYC, p53‐mutant breast and colon cancers, PDCD11, SKP2, ubiquitination

## Abstract

C‐MYC is a proto‐oncoprotein whose dysregulation triggers tumorigenesis and tumor progression in ≈70% of cancer cases. It is presently demonstrated that aberrantly upregulated MYC is caused by the overexpressed and “extra‐nucleolar” PDCD11 in p53‐mutant breast and colon cancer cells, which is highly correlated to tumor progression, metastasis, and recurrence. In the nucleoplasm, PDCD11 binds to the TAD of C‐MYC to prevent SKP2, a transcriptional target of C‐MYC as well as one of the major E3 ligase components targeting C‐MYC, from interacting with and ubiquitinating C‐MYC in feedback. The ensuing stabilized C‐MYC activates downstream signaling to facilitate the cellular G1/S transition, proliferation, and migration. PDCD11 silencing restores SKP2‐mediated C‐MYC degradation, thereby remarkably suppressing tumor growth and metastasis in nude mice. These findings highlight PDCD11 as a novel C‐MYC partner and thereby offer a potential therapeutic rationale to challenge PDCD11‐mediated “pro‐stabilization” effect on C‐MYC, a widely considered “undruggable” target, to combat C‐MYC‐driven malignancies with p53 mutation.

## Graphical abstract

PDCD11 overexpression highly correlates to poor prognosis in p53‐mutant breast and colon cancers. Through interaction with C‐MYC in the nucleoplasm, PDCD11 hinders C‐MYC‐induced SKP2 from binding to the C‐MYC MB2 domain for ubiquitination, thereby stabilizing C‐MYC to drive tumor progression. PDCD11 silencing restores SKP2‐mediated ubiquitination to degrade C‐MYC, offering a potential strategy against C‐MYC‐driven malignancies with p53 mutation.

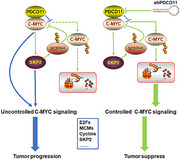

## 1 Introduction

C‐MYC is a basic helix‐loop‐helix leucine zipper transcription factor possessing a N‐terminal transactivation domain (TAD) and a C‐terminal DNA‐binding domain (bHLH‐LZ) essential to its stability and oncogenic transformation.^[^ ^1^ ^]^ Through dimerization with MAX, C‐MYC binds to over 15% of the promoters in the human genome to modulate global gene expression associated with various cellular processes including proliferation, mobility, differentiation, metabolism, apoptosis, and immunomodulation.^[^ ^2^ ^]^ As a necessary and sufficient driver for entry to the DNA synthesis (S) phase, the C‐MYC protein has an extremely short half‐life (≈30 min) in normal cells to avoid triggering uncontrolled proliferation.^[^ ^3^ ^]^ However, its expression and stability are enhanced by alterations in oncogenes such as RAS and RAF, tumor suppressors such as p53 and PTEN, E3 ligase components such as SKP2 and FBXW7, and a panel of phosphorylases and deubiquitinating enzymes (DUBs).^[^ ^4^ ^]^ These multitudinous factors result in elevated levels of the C‐MYC oncoprotein to facilitate tumor development, occurrence, and metastasis in ≈70% of cancer cases.

C‐MYC turnover is mainly controlled by the ubiquitin‐proteasome system, in which the F‐box proteins SKP2 and FBXW7 are the mostly reported E3 subunits that ubiquitinate and destabilize C‐MYC.^[^ ^5^ ^]^ In a manner dependent on GSK3β‐mediated T58 phosphorylation of C‐MYC, FBXW7 binds to the MB1 domain of C‐MYC and thereby contributes to its degradation, whereas dysregulation of C‐MYC phosphorylation or FBXW7 loss of function increases the C‐MYC abundance to promote tumorigenesis.^[^ ^1^, ^2^, ^4^, ^6^ ^]^ In contrast to FBXW7, SKP2 is a canonical transcriptional target of C‐MYC, which ubiquitinates C‐MYC for proteasomal degradation in feedback by binding to either the MB2 or bHLH‐LZ domain of C‐MYC in a phosphorylation‐independent manner.^[^ ^7^ ^]^ By inhibiting SKP2‐mediated ubiquitination, the hepatitis B virus (HBV) X protein enhances C‐MYC stability and contributes to oncogenesis in HBV‐infected cells.^[^ ^8^ ^]^ SKP2 also serves as a C‐MYC downstream effector to facilitate G1/S transition by degrading p27, or as a C‐MYC cofactor to activate other C‐MYC targets.^[^ ^7^, ^9^ ^]^ In some cancer cells uninfected by HBV, C‐MYC is also highly stable and triggers the expression of SKP2 and other targets to accelerate tumor progression,^[^ ^9^, ^10^ ^]^ but it remains unclear how C‐MYC escapes from SKP2‐mediated degradation while the C‐MYC‐SKP2 axis is persistently activated.

To address this issue, we defined the nucleolar protein PDCD11 as a protector and activator of C‐MYC by antagonizing SKP2‐mediated negative feedback regulation in a cell type‐specific manner. In our previous study, PDCD11 was confirmed to be overexpressed in p53‐wild‐type (p53‐WT) colon cancer cells with an “extra‐nucleolar” distribution, which facilitated the recruitment of p53 to HDM2, thereby destroying p53 to accelerate G2/M transition and tumor growth.^[^ ^11^ ^]^ Here, the expression and distribution patterns of PDCD11 did not change in p53‐mutant breast and colon cancer cells, but switched to initiate C‐MYC‐regulated transcription. Through its interaction with the C‐MYC protein in the nucleoplasm, PDCD11 competitively inhibits SKP2 from binding to the C‐MYC MB2 domain for ubiquitination, thereby stabilizing C‐MYC and triggering oncogenic signaling to facilitate G1/S transition, proliferation, and metastasis. PDCD11 silencing decreased the levels of the C‐MYC oncoprotein and its target genes, including SKP2, leading to suppressed tumor progression. Thus, we developed a model showing how PDCD11 helps C‐MYC resist SKP2‐mediated ubiquitination to maintain its high protein levels and oncogenic activity in cancer cells with p53 mutation, which highlights PDCD11 as a potential therapeutic target aimed at destroying “undruggable” C‐MYC^[^ ^4c^ ^]^ for cancer treatment.

## 2 Results

### 2.1 High‐Level PDCD11 Accelerates Progression of p53‐Mutant Breast and Colon Malignancies

**Figure 1:**
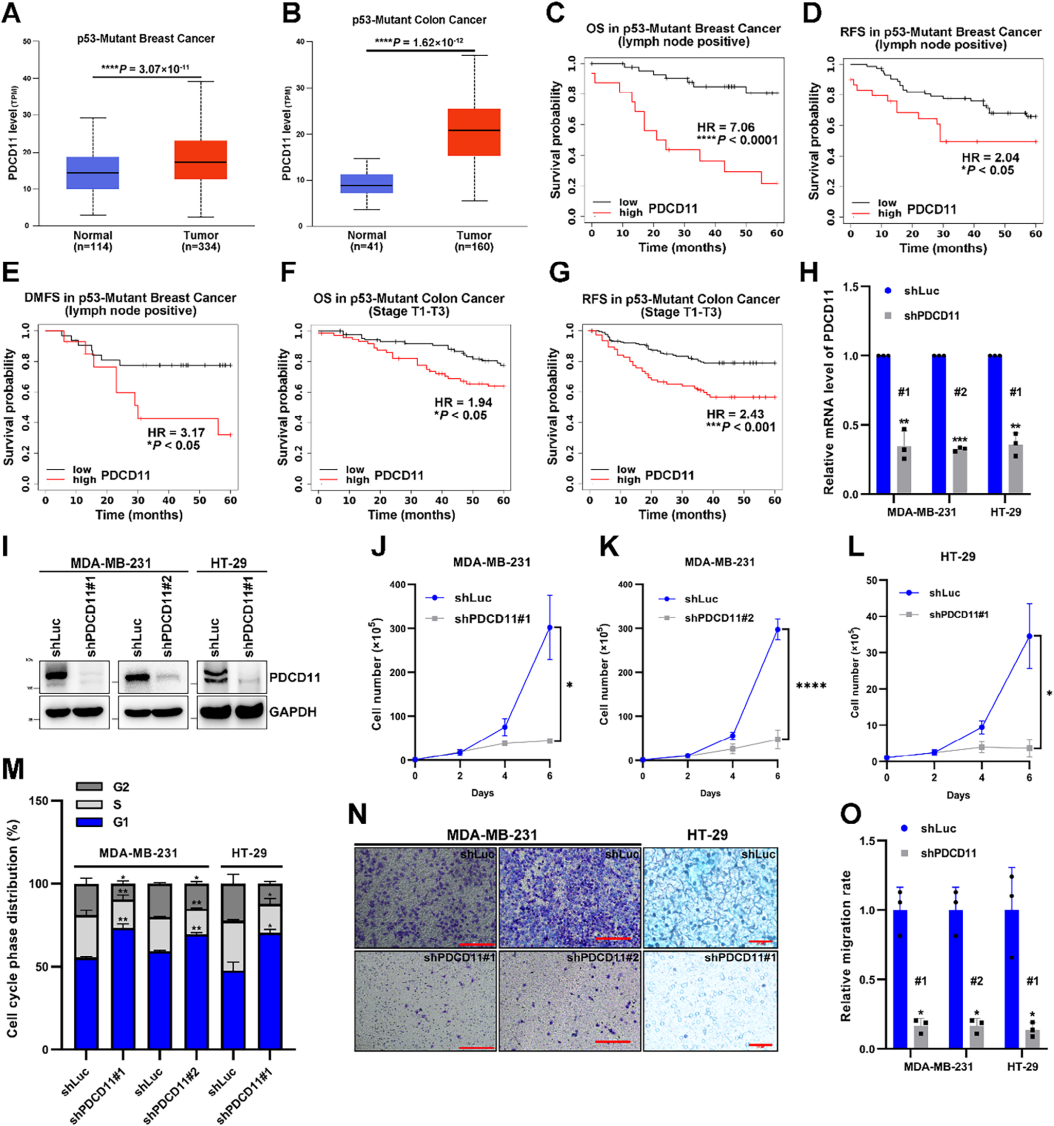
PDCD11 accelerates tumor progression in p53‐mutant breast and colon cancers. A,B) PDCD11 transcriptional levels were compared in normal tissues versus in p53‐mutant tumors. Data were from TCGA database and analyzed using UALCAN. C–G) Kaplan–Meier plotter was used to reveal the correlation between the PDCD11 level and probability of 5‐year OS (overall survival), RFS (recurrence‐free survival), or DMFS (distant metastasis‐free survival) in p53‐mutant breast and colon cancers. HR (> 1) indicates increased risk of tumor progression. A–G) ^*^ *p* < 0.05; ^**^ *p* < 0.01; ^***^ *p* < 0.001; ^****^ *p* < 0.0001 denote significant difference; NS denotes no significance. H–O) All the lentivirus‐transduced cells were treated with Doxy to induce the expression of shLuc, shPDCD11#1, or shPDCD11#2. H) Knockdown efficiency of PDCD11 mRNA was assessed by qRT‐PCR. GAPDH was used as internal control to normalize the values. The normalized values of the cells expressing shLuc were set to 1. I) Cellular levels of PDCD11 protein were assessed by western blotting. GAPDH was used as loading control. J–L) Cell growth curves during the treatment of Doxy. M) Distribution of cell cycle phases was determined by PI staining assay. N) Cell migration was assessed by transwell assay. Bars: 50 µm. O) The migration rates were shown and the mean values of the cells expressing shLuc were set to 1. H,J–M,O) Data are shown as mean ± SD (*n* = 3). ^*^ *p* < 0.05; ^**^ *p* < 0.01; ^***^ *p* < 0.001; ^****^ *p* < 0.0001 denote significant difference; NS denotes no significance.

PDCD11 was previously confirmed to be an oncoprotein that is overexpressed in p53‐WT colon cancer cells,^[^ ^11^ ^]^ whereas its role in p53‐mutant cancers remains unclear. To clarify this, we performed The Cancer Genome Atlas (TCGA) analyses using the University of Alabama at Birmingham Cancer Data Analysis Portal (UALCAN)^[^ ^12^ ^]^ and found higher PDCD11 levels in most of the p53‐mutant cancer types than in normal tissues (Figure S1, Supporting Information). In ≈60% of these cancer types represented by breast cancer (BRCA), intratumor levels of PDCD11 increased on comparing p53‐mutant with p53‐WT, but were unchanged in the other 40% cancer types represented by colon cancer (COAD) (**Figure** **1**A,B; Figure S1, Supporting Information). To investigate the role of PDCD11 overexpression in representative p53‐mutant cancer cases, we performed Kaplan‐Meier survival analyses.^[^ ^13^ ^]^ High‐level PDCD11 shortened the overall survival (OS), recurrence‐free survival (RFS), and distant metastasis‐free survival (DMFS) in patients with p53‐mutant breast cancer with lymph node positivity within 5 years (Figure 1C–E), as well as the 5‐year OS and RFS of p53‐mutant colon cancer patients (Stage T1‐T3) (Figure 1F,G), revealing that PDCD11 abundance is closely and positively correlated.

To verify the oncogenic functions of PDCD11 in representative p53‐mutant cancer cells, we generated MDA‐MB‐231 breast cancer and HT‐29 colon cancer cells that were stably transduced with lentiviral shLuc, shPDCD11#1, or shPDCD11#2. Upon doxycycline (Doxy) treatment, the expression of either shPDCD11#1 or shPDCD11#2 was induced to effectively downregulate PDCD11 at either the mRNA (*p* < 0.01) or protein level (Figure 1H,I), thereby decreasing the proliferation rates of the two cells (Figure 1J–L). To investigate whether PDCD11 regulates proliferation by affecting cell cycle progression, we performed propidium iodide (PI) staining assay and found an increase in the percentage of the G1 population (*p* < 0.05) and a decrease in the percentage of the S population (*p* < 0.05) in cells expressing either shPDCD11#1 or shPDCD11#2, indicating a typical G1/S arrest upon PDCD11 silencing (Figure 1M). Considering that the Kaplan‐Meier survival curves revealed the correlation between PDCD11 levels and tumor metastasis, we performed a transwell assay to verify this. PDCD11 silencing markedly reduced the migration rates of MDA‐MB‐231 and HT‐29 cells (*p* < 0.05) (Figure 1N,O), indicating that PDCD11 promotes metastasis by regulating cancer cell migration.

Thus, high levels of PDCD11 indicate poor prognosis in p53‐mutant breast and colon cancers. By facilitating the G1/S transition and migration, PDCD11 functions as an oncoprotein to accelerate cancer cell growth and metastasis.

### 2.2 PDCD11 Promotes Cancer Cell Growth and Migration by Activating C‐MYC Signaling

To investigate whether PDCD11 promotes cell growth and migration by regulating the levels of mutant p53, we performed western blot analyses. Unlike in HCT116 cells with WT p53,^[^ ^11^ ^]^ PDCD11 lost its capacity to regulate p53‐R280K due to the low HDM2 level in MDA‐MB‐231 cells^[^ ^14^ ^]^ but showed a greatly enhanced affinity for p53‐R273H to negatively regulate this mutant in HT‐29 cells^[^ ^15^ ^]^ (Figures S2 and S3A,B, Supporting Information). Considering that p53‐R273H usually functions as an oncoprotein to elicit oncogenic phenotypes,^[^ ^16^ ^]^ it is unlikely that PDCD11 promotes tumor progression by downregulating this mutant.

**Figure 2:**
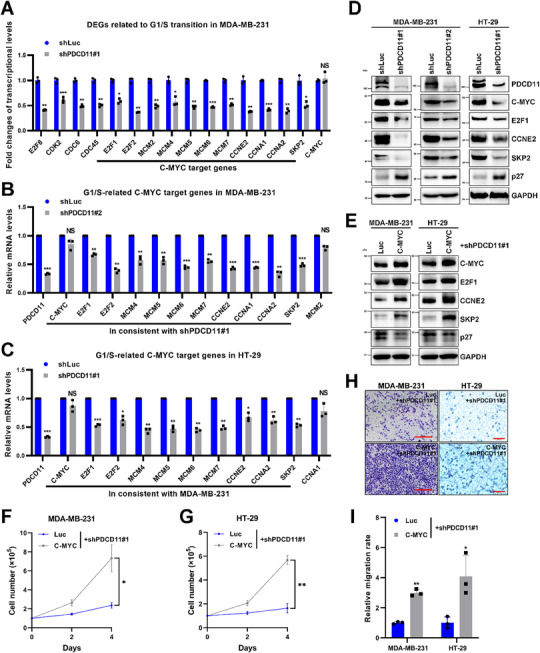
PDCD11 facilitates cancer cell proliferation and migration by upregulating the C‐MYC oncoprotein and its target genes. All the lentivirus‐transduced cells were treated with Doxy to induce the expression of shRNAs. A) RNA‐seq analysis was performed to screen the transcripts regulated by PDCD11 (*n* = 3). Fold changes of transcriptional levels of the key DEGs related to G1/S transition were shown and the C‐MYC target genes were marked. B,C) qRT‐PCR analyses were performed to assess the mRNA levels of PDCD11, C‐MYC, and G1/S‐related C‐MYC target genes in PDCD11‐silenced cells. GAPDH was used as internal control to normalize the values. The normalized values of the cells expressing shLuc were set to 1. D,E) WB analyses were performed to assess the levels of PDCD11, C‐MYC, E2F1, CCNE2, SKP2, and p27 proteins in the cells with or without transfection of pcDNA3‐Luc or pcDNA3‐C‐MYC. GAPDH was used as loading control. F and G) Growth curves of PDCD11‐silenced cells transfected with pcDNA3 plasmids. H) Transwell assay was performed to assess cell migration. Bars: 50 µm. I) The migration rates were shown and the mean values of the cells transfected with pcDNA3‐Luc were set to 1. A–C,F,G,I) Data are shown as mean ± SD. ^*^ *p* < 0.05; ^**^ *p* < 0.01; ^***^ *p* < 0.001; ^****^ *p* < 0.0001 denote significant difference; NS denotes no significance.

To explore how PDCD11 exerts the oncogenic functions, we performed RNA‐seq analysis and found significant alternations in the transcriptional profile of PDCD11‐silenced MDA‐MB‐231 cells (Figure S4A–C, Supporting Information). Consistent with the results of TCGA, Kaplan‐Meier analyses, and oncogenic phenotype assays (Figure 1), Reactome enrichment and Disease Ontology (DO) enrichment analyses revealed that PDCD11 promotes tumorigenesis and tumor progression by regulating G1/S transition during cell cycle progression (Figure S4D,E, Supporting Information). Moreover, most of the downregulated G1/S‐related differentially expressed genes (DEGs) in MDA‐MB‐231 cells expressing shPDCD11#1 are reported C‐MYC target genes including E2F1/2, MCM2, MCM4‐7, CCNE2, CCNA1/2, and SKP2^[^ ^17^ ^]^ (**Figure** **2**A), whose transcriptional levels were also confirmed to be reduced in the cells expressing shPDCD11#2 (excluding MCM2) and in PDCD11‐silenced HT‐29 cells (excluding CCNA1) through qRT‐PCR analyses (Figure 2B,C). Thus, PDCD11 upregulates G1/S‐related C‐MYC targets. We also assessed the transcriptional level of EGR1, a noncanonical C‐MYC target related to apoptosis.^[^ ^18^ ^]^ In contrast, PDCD11 silencing led to an increased but not decreased EGR1 level, indicating that this apoptosis‐promoting gene was not upregulated by PDCD11 (Figure S4F, Supporting Information). The clinical correlation between the levels of PDCD11 and most of these G1/S‐related C‐MYC targets was further confirmed through TCGA‐based Pearson analysis using Gene Expression Profiling Interactive Analysis 2 (GEPIA2)^[^ ^19^ ^]^ (Figure S5, Supporting Information), suggesting that PDCD11 promotes tumor progression by modulating C‐MYC signaling.

Since PDCD11 regulated the transcriptional levels of C‐MYC target genes, but not C‐MYC itself (Figure 2A–C), we speculated that PDCD11 might affect the level of C‐MYC protein. To verify this, we performed western blot (WB) analyses and found that PDCD11 silencing decreased the protein levels of either C‐MYC or its representative oncogenic targets including E2F1, CCNE2, and SKP2 (Figure 2D), which are essential drivers for tumor growth and metastasis.^[^ ^19^, ^20^ ^]^ Therefore, downregulation of SKP2 increased the levels of p27 protein^[^ ^9a^ ^]^ (Figure 2D), contributing to the G1/S arrest induced by PDCD11 silencing (Figure 1M). To further verify the oncogenic effect of the PDCD11‐C‐MYC axis, we complemented the C‐MYC abundance in PDCD11‐silenced cells. Overexpression of C‐MYC increased the levels of E2F1, CCNE2, and SKP2, and thus decreased the levels of p27 (Figure 2E) to rescue the arrested cell growth and migration (Figure 2F–I), indicating that PDCD11 elicits the oncogenic phenotypes of p53‐mutant cancer cells through the upregulation of C‐MYC downstream pathways. We also assessed the mRNA and protein levels of C‐MYC in HCT116 colon cancer cells harboring WT p53. PDCD11 silencing increased the level of p53 protein but did not affect the level of C‐MYC mRNA or protein, indicating that the PDCD11‐C‐MYC axis does not function in the p53‐WT cells (Figure S6A,B, Supporting Information).

Thus, PDCD11 upregulates C‐MYC protein but not C‐MYC mRNA to activate downstream effector molecules including but not limited to E2F1, CCNE2, and SKP2, thereby exerting its tumor‐promoting functions.

### 2.3 PDCD11 Stabilizes C‐MYC Oncoprotein by Resisting SKP2‐Mediated Ubiquitination

**Figure 3:**
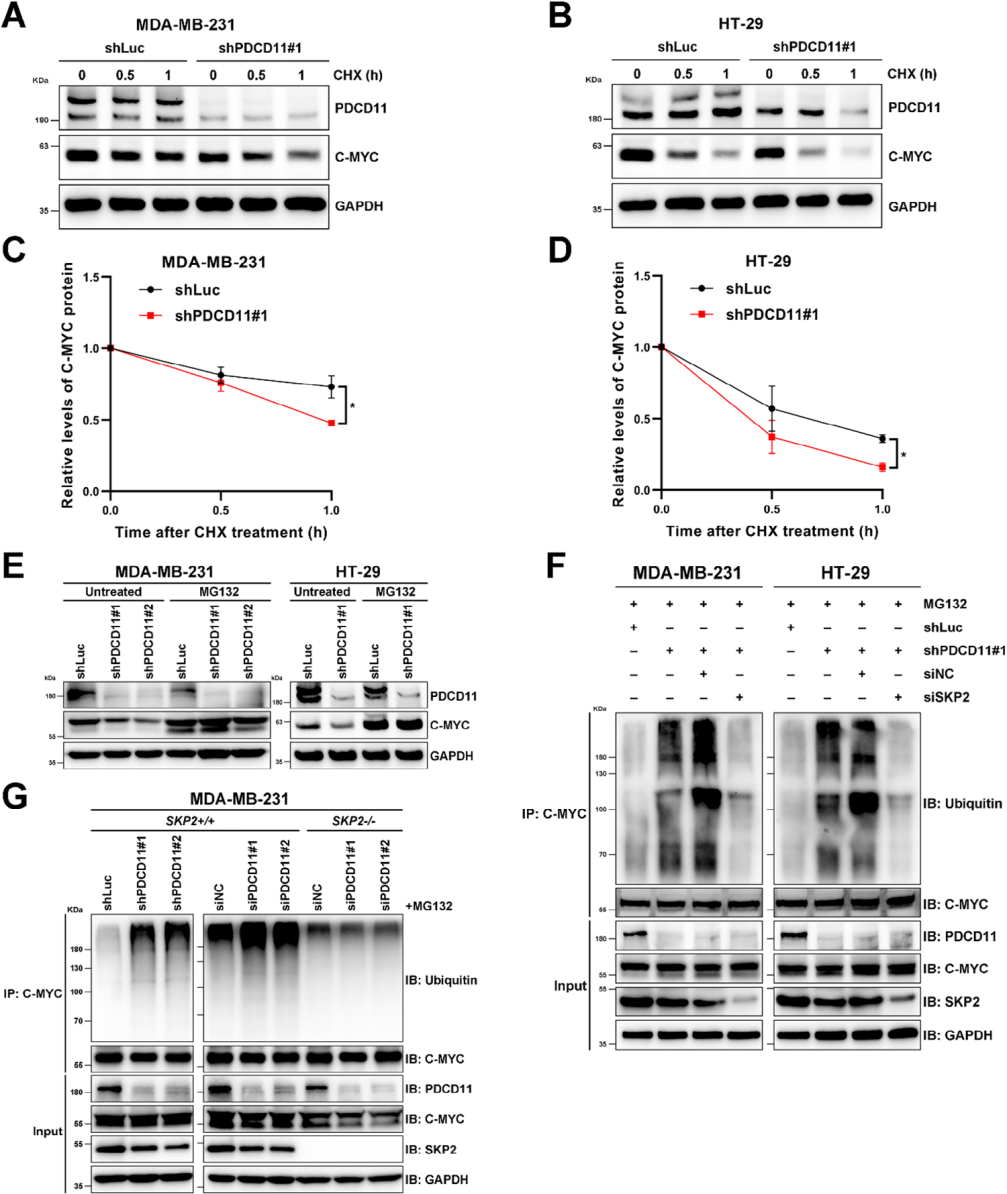
PDCD11 stabilizes C‐MYC via attenuating the SKP2‐dependent ubiquitination of C‐MYC. All the lentivirus‐transduced cells were treated with Doxy to induce the expression of shRNAs. A,B) WB analyses were performed to assess the levels of PDCD11 and C‐MYC proteins in the cells treated with 50 µg mL^−1^ CHX for indicated times. GAPDH was used as loading control. C,D) Relative quantification of C‐MYC protein levels was shown (*n* = 3). The C‐MYC levels at 0 h were set to 1. Data are shown as mean ± SD. ^*^ *p* < 0.05; ^**^ *p* < 0.01; ^***^ *p* < 0.001; ^****^ *p* < 0.0001 denote significant difference; NS denotes no significance. E) WB analyses were performed to assess the levels of PDCD11 and C‐MYC proteins in the cells untreated or treated with 20 µm MG132 for 8 h. GAPDH was used as loading control. F,G) The cells were untransfected or transfected with siRNAs, followed by treatment with MG132 (20 µm, 8 h) prior to be harvested. Ubiquitinated and nonubiquitinated C‐MYC was immunoprecipitated using anti‐C‐MYC from the cells. Anti‐Ubiquitin and anti‐C‐MYC were used to detect ubiquitinated and nonubiquitinated C‐MYC, respectively. The levels of C‐MYC, SKP2, and PDCD11 proteins in the input cell lysate were determined by western blotting. GAPDH was used as loading control.

To investigate whether PDCD11 regulates C‐MYC oncoprotein by affecting its stability, we performed the cycloheximide (CHX) chase assay.^[^ ^21^ ^]^ PDCD11 silencing remarkably accelerated the degradation rate of C‐MYC in either MDA‐MB‐231 or HT‐29 cells (**Figure** **3**A–D), indicating that PDCD11 stabilizes C‐MYC to increase its protein level.

Considering that C‐MYC stability is primarily affected by the ubiquitin‐proteasome system, we treated the cells with the proteasome inhibitor MG132.^[^ ^21^ ^]^ Unlike in untreated cells, PDCD11 silencing no longer regulated the levels of C‐MYC protein when the proteasomal pathway was blocked by MG132 (Figure 3E), indicating that PDCD11 regulates C‐MYC through this pathway. Therefore, we performed an immunoprecipitation (IP)‐ubiquitination assay^[^ ^22^ ^]^ to assess the ubiquitination level of C‐MYC in MG132‐treated cells. PDCD11 silencing increased the levels of ubiquitinated C‐MYC in the cells (Figure 3F,G), indicating that PDCD11 stabilizes C‐MYC by inhibiting its ubiquitination. Considering that SKP2 and FBXW7 are the two major E3 subunits that ubiquitinate C‐MYC,^[^ ^5^ ^]^ we transfected siRNAs targeting these two genes into PDCD11‐silenced cells. The siSKP2 noticeably reduced the levels of ubiquitinated C‐MYC (Figure 3F), whereas siFBXW7 did not (Figure S7, Supporting Information), suggesting that PDCD11 stabilizes C‐MYC by inhibiting SKP2‐mediated but not FBXW7‐mediated ubiquitination. We also constructed MDA‐MB‐231*^SKP2‐/−^* cells (Figure S8, Supporting Information) and found that PDCD11 silencing no longer increased the level of ubiquitinated C‐MYC after SKP2 knockout (Figure 3G), confirming that PDCD11 enhances C‐MYC stability in a SKP2‐dependent manner.

Thus, PDCD11 helps C‐MYC resist SKP2‐mediated ubiquitination in p53‐mutant cancer cells, thereby protecting this oncoprotein from proteasomal degradation.

### 2.4 “Extra‐Nucleolar” PDCD11 Binds to C‐MYC Transactivation Domain that Comprises the SKP2‐Interacting Site

**Figure 4:**
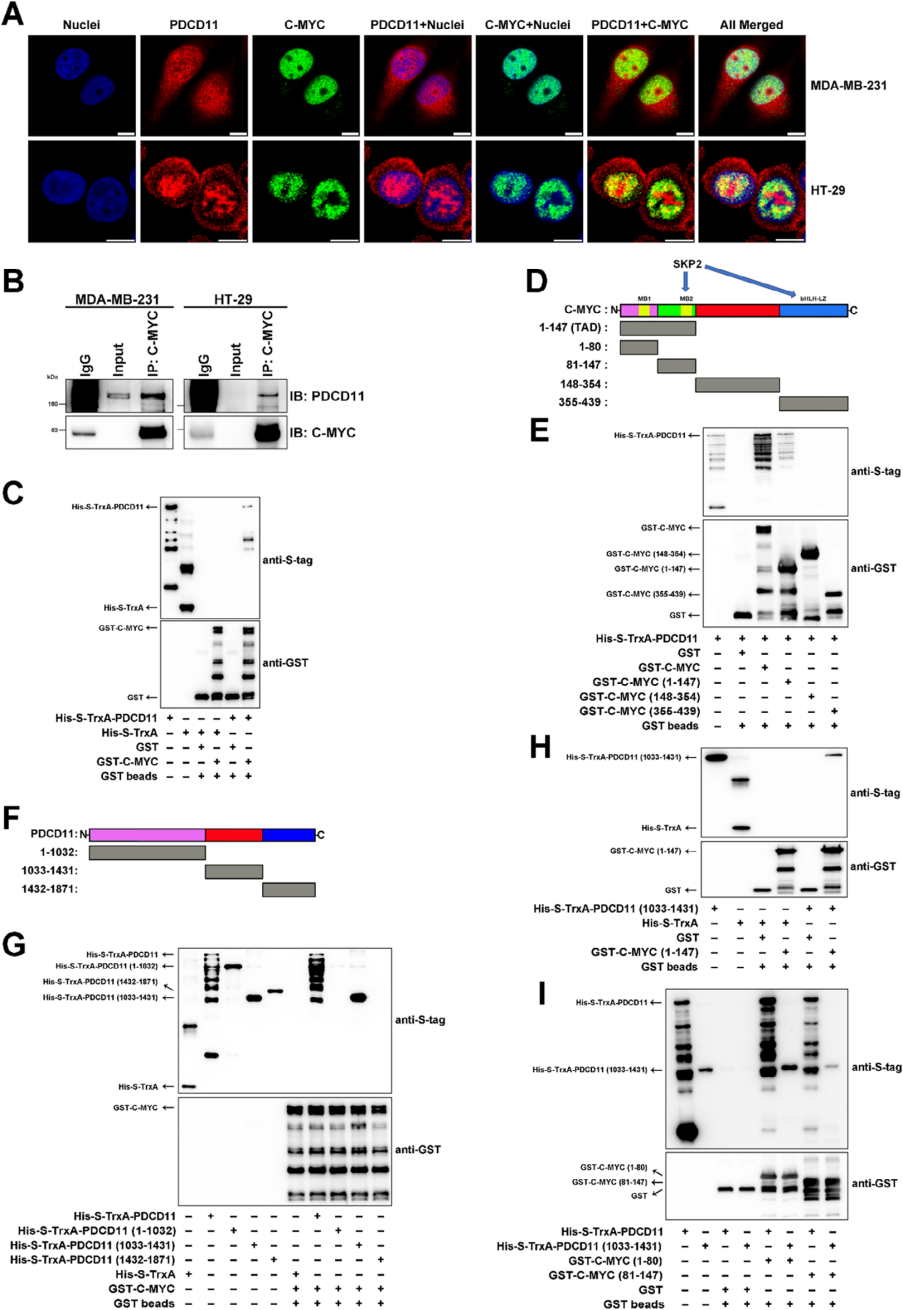
Nucleoplasmic PDCD11 binds to the C‐MYC TAD through its aa 1033–1431 region. A) IF assay was performed to assess the subcellular localization of PDCD11 (red), C‐MYC (green), and their colocalization (yellow) in the cells. The nuclei were stained blue. Scale bars: 10 µm. B) Co‐IP assay was performed to assess the PDCD11‐C‐MYC interaction in vivo using anti‐C‐MYC. Normal rabbit IgG was used as negative control. Input represents 5% of the cell lysate utilized for IP. C‐I) GST pulldown assay was performed to assess the PDCD11‐C‐MYC interaction in vitro and identify the binding regions. C) Full‐length (PDCD11‐FL) interacts with C‐MYC‐FL. D) A schematic diagram showing the construction of C‐MYC truncations. E) PDCD11‐FL interacts with C‐MYC (1–147). F) A schematic diagram showing the construction of PDCD11 truncations. G) PDCD11 (1033–1431) interacts with C‐MYC‐FL. H) PDCD11 (1033–1431) interacts with C‐MYC (1–147). I) Either PDCD11‐FL or PDCD11 (1033–1431) interacts with either C‐MYC (1–80) and C‐MYC (81–147).

The key problem to be solved in this study was how the decreased levels of SKP2 by PDCD11 silencing effectively ubiquitinated C‐MYC (Figure 3F,G). Considering that PDCD11 exhibits an “extra‐nucleolar” distribution in p53‐WT colon cancer cells^[^ ^11^ ^]^ and C‐MYC is widely considered a nucleoprotein in a noncell type‐specific manner,^[^ ^23^ ^]^ we speculated that PDCD11 and C‐MYC might be colocalized to form a complex in the nucleus of p53‐mutant cancer cells, thereby affecting reported intranuclear C‐MYC‐SKP2 interaction.^[^ ^7a^ ^]^ We first performed HitPredict analysis^[^ ^24^ ^]^ and identified C‐MYC as a highly probable binding partner with PDCD11, as confirmed by the results of five independent high‐throughput experiments (Table S1, Supporting Information). Follow‐up immunofluorescence (IF) and co‐IP assays indicated that PDCD11 was not confined to the nucleolus but also to the nucleoplasm and cytoplasm of p53‐mutant MDA‐MB‐231 and HT‐29 cells, thereby colocalizing with C‐MYC to form a complex in the nucleoplasm but not in the nucleolus (**Figure** **4**A,B). Furthermore, we performed GST‐pulldown assay and confirmed that PDCD11 directly bound to C‐MYC (Figure 4C; Figure S3A,C, Supporting Information).

To identify the C‐MYC‐binding region on PDCD11 and the PDCD11‐binding region on C‐MYC, we constructed a panel of truncated variants of the two proteins (Figure 4D,F; Figure S3A,C, Supporting Information). GST pulldown analyses showed that PDCD11 bound to the TAD of C‐MYC through its aa 1033–1431 region (Figure 4D–H). To identify a more precise region of the N‐terminal C‐MYC for PDCD11 binding, we divided the C‐MYC TAD into an aa 1–80 region with the MB1 domain (aa 45–63) and an aa 81–147 region with the reported SKP2‐binding domain MB2 (aa 128–143)^[^ ^1b^ ^]^ (Figure 4D; Figure S3D, Supporting Information). Both regions were confirmed to be the binding partners of PDCD11 (Figure 4I).

Thus, PDCD11 colocalizes with the C‐MYC oncoprotein in the nucleoplasm of p53‐mutant cancer cells, thereby forming a complex by binding to C‐MYC TAD, which comprises the SKP2‐binding site.

### 2.5 PDCD11 Hinders SKP2 from Interacting with and Ubiquitinating C‐MYC

**Figure 5:**
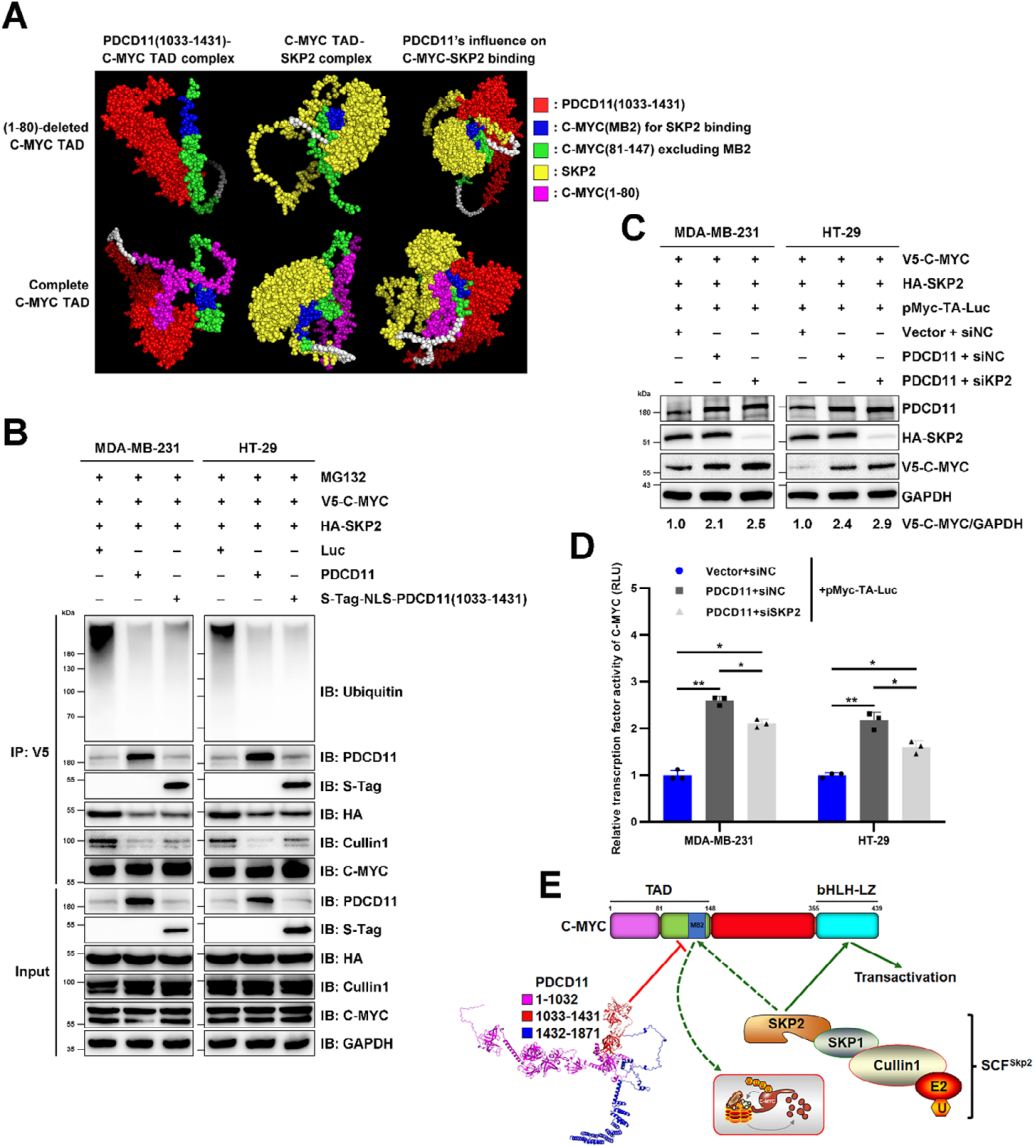
PDCD11 attenuates C‐MYC ubiquitination by competitively hindering the C‐MYC‐SKP2 interaction but retains the transcription factor activity of C‐MYC. A) ColabFold was used to predict the structure of PDCD11 (1033–1431)‐C‐MYC (81–147/1–147), C‐MYC (81–147/1–147)‐SKP2 complexes, as well as the influence of PDCD11 (1033–1431) on the formation of C‐MYC (81–147/1–147)‐SKP2 complexes. B–D) All the cells were transduced with lentiviruses for the stable coexpression of V5‐C‐MYC and HA‐SKP2. B) Ubiquitinated and nonubiquitinated V5‐C‐MYC was immunoprecipitated using anti‐V5 from the cells treated with MG132 (20 µm, 8h). Anti‐Ubiquitin, anti‐C‐MYC, anti‐PDCD11, anti‐S‐tag, anti‐HA, and anti‐Cullin1 were used to detect ubiquitinated and nonubiquitinated V5‐C‐MYC, co‐immunoprecipitated PDCD11, S‐Tag‐NLS‐PDCD11 (1033–1431), HA‐SKP2, and Cullin1 proteins. The levels of V5‐C‐MYC, PDCD11, S‐Tag‐NLS‐PDCD11 (1033–1431), HA‐SKP2, and Cullin1 proteins in the input cell lysate were determined by western blotting. GAPDH was used as loading control. C,D) Cells were cotransfected with pcDNA3‐Vector/PDCD11, siNC/siSKP2, and pMyc‐TA‐Luc. C) WB analyses were performed to assess the levels of PDCD11, V5‐C‐MYC, and HA‐SKP2 proteins. GAPDH was used as loading control. Fold changes of the C‐MYC level were shown and the values of the cells cotransfected with siNC and pcDNA3‐Vector were set to 1. D) Firefly luciferase reporter gene assay was performed to measure the relative Luc luminescence (RLU), which indicates the TFA of C‐MYC in the cells. The normalized mean values of the cells cotransfected with pcDNA3‐Vector, siNC, and pMyc‐TA‐Luc were set to 1. Data are shown as mean ± SD. ^*^ *p* < 0.05; ^**^ *p* < 0.01; ^***^ *p* < 0.001; ^****^ *p* < 0.0001 denote significant difference; NS denotes no significance. E) A model showing that PDCD11 attenuates C‐MYC ubiquitination by blocking the binding of SCF^SKP2^ to the MB2 domain, but still allows SKP2 to bind to the bHLH‐LZ domain of C‐MYC to retain its TFA.

To verify whether the PDCD11‐binding site on the C‐MYC TAD overlapped with the SKP2‐binding site, we performed a ColabFold analysis based on the AlphaFold2 AI system.^[^ ^2^, ^25^ ^]^ As shown by the predicted structural models (**Figure** **5**A), PDCD11 (1033–1431) binds to the MB2 domain (aa 128–143), a defined SKP2 binding site on C‐MYC,^[^ ^7a^ ^]^ when forming a complex with the complete C‐MYC TAD. Deletion of aa 1–80 from TAD deprived the MB2‐binding capacity of PDCD11, although it could still bind to the MB2‐excluded region of C‐MYC (81–147) (Figure 5A). These results revealed that C‐MYC (1–80) is essential to motivate PDCD11 and C‐MYC to form a stable complex, which might help PDCD11 (1033–1431) occupy the MB2 domain of C‐MYC to antagonize SKP2 binding (Figure 5A).

Since SKP2 recruits SKP1 and Cullin1 to form an E3 ligase complex (SCF^SKP2^) to ubiquitinate the substrate,^[^ ^7a^ ^]^ we hypothesized that PDCD11 competitively hinders SKP2 from binding to the C‐MYC MB2 domain, thereby protecting C‐MYC from recognition by the SCF^SKP2^ complex for ubiquitination and degradation (Figure 5E). Therefore, we performed competitive co‐IP and ubiquitination assays by overexpressing PDCD11 and its truncated C‐MYC‐binding region (aa 1033–1431) in MDA‐MB‐231 and HT‐29 cells stably expressing V5‐C‐MYC and HA‐SKP2. Due to the nucleoplasmic localization of PDCD11‐C‐MYC complex (Figure 4A,B), PDCD11 (1033–1431) was fused with a previously verified nuclear localization sequence (NLS).^[^ ^11^ ^]^ Introducing either PDCD11 or S‐TAG‐NLS‐PDCD11 (1033–1431) inhibited V5‐C‐MYC from interacting with either HA‐SKP2 or Cullin1 (Figure 5B). Therefore, this attenuated SCF^SKP2^‐mediated ubiquitination to stabilize C‐MYC (Figure 5B), providing strong evidence for our hypothesis.

Considering the likely overlapping binding sites on PDCD11 (1033–1431) with HDM2 (aa 349–497)^[^ ^11^ ^]^ and C‐MYC (Figure 4), these data also help to explain why PDCD11 loses its capacity to regulate the C‐MYC level in p53‐WT cells (Figure S6A,B, Supporting Information). As shown by the AlphaFold2‐predicted models, the high level of HDM2 induced by WT p53^[^ ^11^ ^]^ competed with C‐MYC to bind to PDCD11 (Figures S2C and S6C, Supporting Information), whereas p53 mutants hardly triggered HDM2 expression^[^ ^26^ ^]^ (Figure S2C, Supporting Information), thereby allowing a stronger PDCD11‐C‐MYC interaction to resist SKP2‐mediated degradation.

In addition to being an E3 subunit, SKP2 also functions as a C‐MYC cofactor to activate other C‐MYC target genes by binding to either the MB2 or the bHLH‐LZ domain of the C‐MYC oncoprotein.^[^ ^7a^ ^]^ To investigate whether attenuation of C‐MYC‐SKP2 interaction by PDCD11 reduces the transcription factor activity (TFA) of C‐MYC, we performed a firefly luciferase reporter gene assay.^[^ ^27^ ^]^ In both MDA‐MB‐231 and HT‐29 cells, overexpression of PDCD11 unsurprisingly upregulated the levels of C‐MYC protein (Figure 5C), leading to a remarkably increased TFA of C‐MYC (*p* < 0.01) (Figure 5D). This is probably because PDCD11 only hinders SKP2 from binding to the N‐terminal MB2 but not the C‐terminal bHLH‐LZ domain on C‐MYC, which enables C‐MYC to retain a sizeable proportion of TFA (Figures 4 and 5E). In support of this speculation, the highly stabilized C‐MYC protein by overexpressed PDCD11 was only slightly upregulated in response to siSKP2 transfection (Figure 5C), but exhibited decreased TFA (*p* < 0.05) (Figure 5D). Considering the higher C‐MYC TFA in SKP2‐silenced and PDCD11‐overexpressed cells than in SKP2‐overexpressed cells with a lower level of PDCD11 (*p* < 0.05) (Figure 5D), we concluded that PDCD11‐promoted C‐MYC stabilization contributes more than SKP2 in enhancing C‐MYC‐regulated transcription.

Thus, PDCD11 hinders SKP2 from binding to the C‐MYC MB2 domain, thereby helping C‐MYC resist SCF^SKP2^‐mediated ubiquitination and degradation. The ensuing upregulated C‐MYC results in increased TFA that activates downstream oncogenic signaling pathways.

### 2.6 PDCD11 Is a Potential Therapeutic Target for p53‐Mutant Breast and Colon Cancers

**Figure 6:**
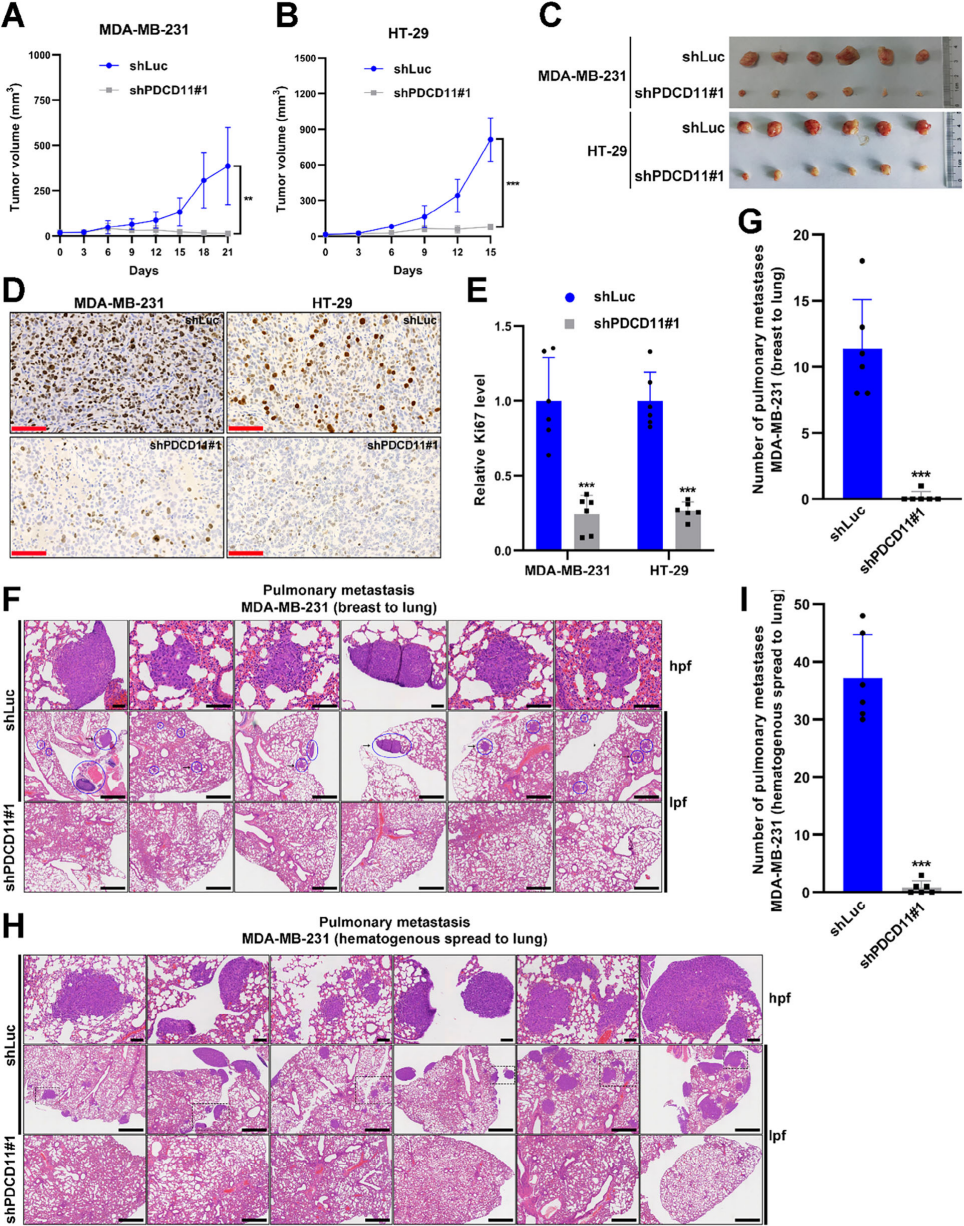
PDCD11 silencing is an efficient therapeutic strategy against p53‐mutant breast and colon malignancies. A–G) Nude mice bearing lentivirus‐transduced MDA‐MB‐231 or HT‐29 tumors were administered with Doxy in drinking water for 21 or 15 days, respectively, to silence the intratumor levels of PDCD11. A) Growth curves of MDA‐MB‐231 tumors seeded in the mammary fat pad (*n* = 6). B) Growth curves of HT‐29 subcutaneous xenografts (*n* = 6). C) Images of the harvested primary tumors at the end of the treatment course. D) Representative photographs for the tumor tissue sections with Ki67 staining (brown). Bars: 100 µm. E) Relative Ki67 levels in the tumors (*n* = 6). The mean values of the tumors expressing shLuc were set to 1. F) Representative photographs of the lung tissue sections with H&E staining (*n* = 6). In the low‐power field (lpf), the metastases were circled in blue and the arrow‐indicated region was magnified in a high‐power field (hpf). Bars: 1 mm (lpf), 100 µm (hpf). G) Numbers of the pulmonary metastases (breast to lung) at day 21 (*n* = 6). H,I) After intravenously injected with lentivirus‐transduced MDA‐MB‐231 cells, nude mice were fed with Doxy for 35 days to induce the expression of shRNAs in the cancer cells. The lungs were harvested for H&E staining analysis. H) Representative images showing the pulmonary metastasis (*n* = 6). The outlined region in the lpf was magnified in a hpf. Bars: 1 mm (lpf), 100 µm (hpf). I) Numbers of the pulmonary metastases (hematogenous spread to lung) at day 35 (*n* = 6). A,B,E,G,I) Data are shown as mean ± SD. ^*^ *p* < 0.05; ^**^ *p* < 0.01; ^***^ *p* < 0.001; ^****^ *p* < 0.0001 denote significant difference; NS denotes no significance.

To determine whether PDCD11 is a potential target for the treatment of p53‐mutant breast and colon cancers, MDA‐MB‐231 and HT‐29 cells stably and inducibly expressing shRNAs were inoculated into nude mice. Consistent with the data obtained in vitro (Figure 1J–L), both the in situ MDA‐MB‐231 tumors in the breast and the subcutaneous HT‐29 tumors expressing shPDCD11 grew much slower than the controls expressing shLuc during Doxy treatment (**Figure** **6**A–C). Until days 21 and 15, PDCD11 silencing led to growth inhibition rates of ≈97% in MDA‐MB‐231‐bearing mice (*p* < 0.01) and ≈90% in HT‐29‐bearing mice (*p* < 0.001), respectively. Additionally, we found that PDCD11 silencing significantly decreased Ki67 levels (*p* < 0.001), a canonical marker indicating tumor cell proliferation,^[^ ^28^ ^]^ in both MDA‐MB‐231 and HT‐29 tumors, further confirming the antitumor efficacy of shPDCD11 (Figure 6D,E).

Considering the high risk of pulmonary metastasis in p53‐mutant breast cancers driven by C‐MYC,^[^ ^29^ ^]^ we collected lung tissues from MDA‐MB‐231‐bearing mice on day 21 for H&E staining according to our previous work.^[^ ^30^ ^]^ Compared to the shLuc control, PDCD11 silencing remarkably reduced the number of pulmonary metastatic nodules originating from the breast tumors (*p* < 0.001) (Figure 6F,G). To assess whether PDCD11 promotes the hematogenous dissemination of cancer cells, shRNA‐transduced MDA‐MB‐231 cells were also intravenously injected into nude mice. After a 35‐day treatment with Doxy, numerous sizeable metastases were observed in the lung tissues of the shLuc group, but rarely in the shPDCD11 group, highlighting the powerful anti‐metastasis efficacy of suppressing PDCD11 expression (Figure 6H,I).

Thus, PDCD11 is a potential therapeutic target against p53‐mutant breast and colon cancers and PDCD11 silencing is a potent therapeutic strategy against tumor growth and metastasis.

## 3 Discussion

C‐MYC, a strongly evidenced oncoprotein, correlates with almost all the hallmarks of cancer and is dysregulated in more than 70% of human malignancies, driving both tumor initiation and maintenance.^[^ ^4^, ^31^ ^]^ Due to the extremely unstable nature of the C‐MYC protein, its half‐life is prolonged by numerous oncogenic factors that regulate diverse posttranslational mechanisms, especially ubiquitination‐related routes.^[^ ^5^, ^32^ ^]^ Among this network, which comprises various E3 ligase components, phosphorylases, and DUBs, SKP2 and FBXW7 are the most studied E3 subunits that directly interact with C‐MYC for ubiquitination. Although several studies have revealed how FBXW7 loses its function in cancer cells,^[^ ^1^, ^2^, ^4^, ^6^ ^]^ the role of the C‐MYC‐SKP2 loop in human cancer remains confused. As a canonical transcriptional target of C‐MYC, SKP2 functions as a p27 inhibitor and C‐MYC co‐factor to facilitate cell growth and survival, but also as an E3 subunit to ubiquitinate and degrade C‐MYC in feedback.^[^ ^7^, ^17^ ^]^ Cancelling either SKP2‐C‐MYC (MB2) or SKP2‐C‐MYC (bHLH‐LZ) interaction deprives most of the capacity of SKP2 to destabilize C‐MYC.^[^ ^7a^ ^]^ Both the HBV X protein and the tumor suppressor ARF can upregulate C‐MYC by blocking SKP2 from binding to the MB2 domain, but resulting in paradoxical outcomes.^[^ ^8^, ^18^ ^]^ Exogenous HBV infection induces C‐MYC‐mediated oncogenesis, whereas endogenous ARF recruits C‐MYC to the promoter of the noncanonical C‐MYC target gene EGR1, thereby triggering cancer cell apoptosis. To date, there is no consensus that C‐MYC‐SKP2 interaction contributes to tumor inhibition or promotion.

**Figure 7:**
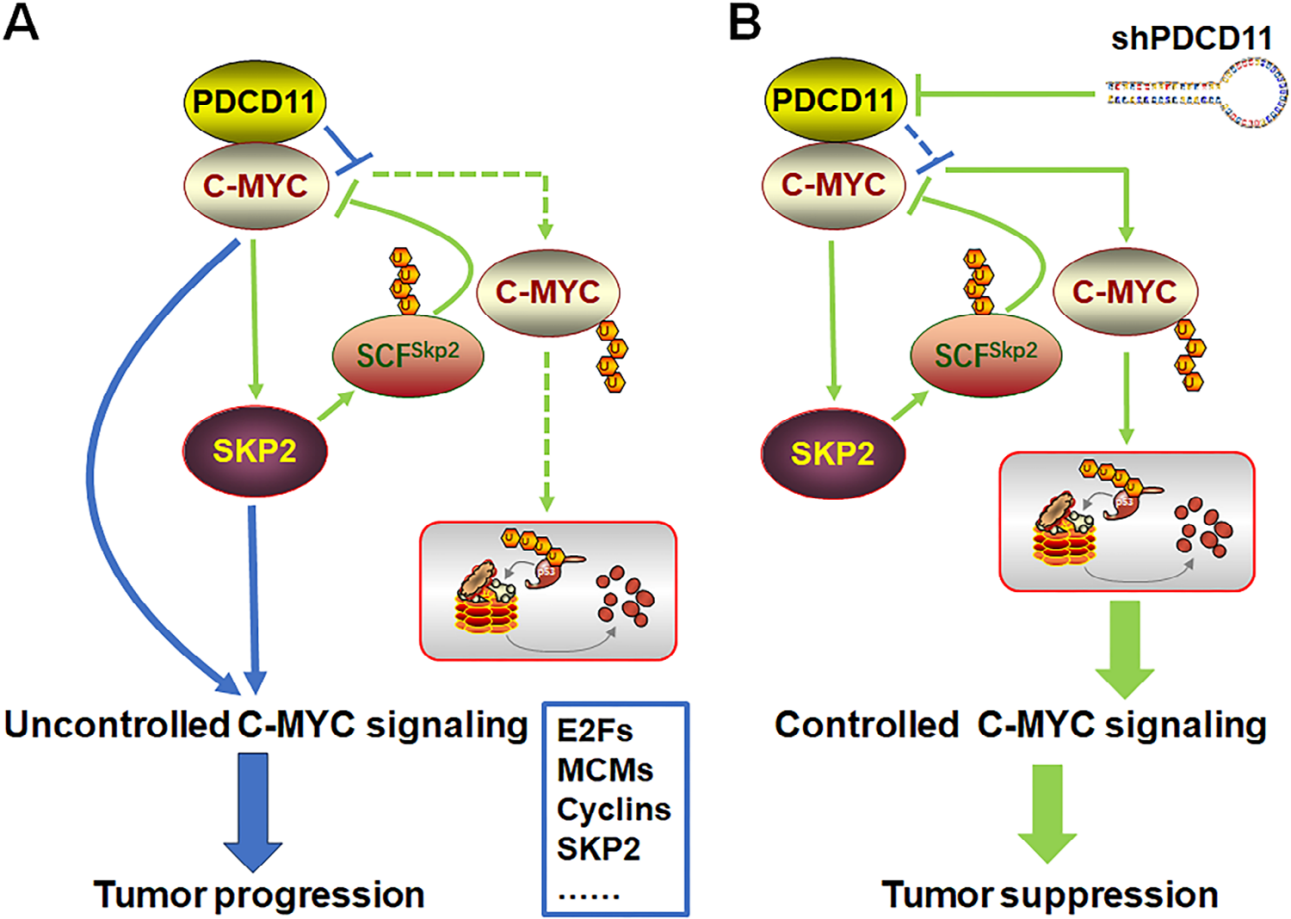
A proposed regulatory model showing how PDCD11 hyperactivates C‐MYC signaling. A) PDCD11 interacts with C‐MYC and thereby hinders SKP2, a transcriptional target of C‐MYC, from ubiquitinating C‐MYC in feedback, leading to a stabilized C‐MYC oncoprotein to accelerate tumor progression. B) PDCD11 silencing restores the C‐MYC‐SKP2 negative feedback loop to degrade C‐MYC, thereby suppressing C‐MYC‐regulated oncogenic signaling.

Helpful to solve this issue, our findings present a hindered C‐MYC‐SKP2 negative feedback loop by PDCD11 (**Figure** **7**). Similar to its expression and distribution patterns in p53‐WT colon cancer cells,^[^ ^11^ ^]^ PDCD11 is also overexpressed and spills over from the nucleolus, thereby colocalizing and interacting with C‐MYC in the nucleoplasm of p53‐mutant breast and colon cancer cells (Figure 4). By antagonizing SKP2‐mediated C‐MYC ubiquitination (Figure 5), PDCD11 disrupts the balance between C‐MYC and SKP2, resulting in uncontrolled C‐MYC signaling and tumor progression (Figure 7A). PDCD11 silencing withdraws the protection from C‐MYC, thereby allowing SKP2 to degrade it in feedback (Figure 3). This helps rebuild the C‐MYC‐SKP2 balance, leading to controlled C‐MYC signaling and tumor suppression (Figure 7B). Accordingly, “extra‐nucleolar” PDCD11 should be defined as a tumor marker indicating an unfavorable prognosis and a potential therapeutic target (Figures 1 and 6), which acts as a C‐MYC partner to drive the development of breast and colon malignancies with p53 mutation.

Our findings also highlight the contribution of the C‐MYC‐SKP2 axis to PDCD11‐driven tumor progression. On one hand, SKP2 was transcriptionally activated by C‐MYC, thereby decreasing p27 to promote G1/S transition and cancer cell growth (Figures 1 and 2). On the other hand, SKP2 helps C‐MYC retain its TFA in PDCD11‐overexpressed cancer cells, which is not weakened by hindered interaction with SKP2. This is mainly because PDCD11 only prevents SKP2 from binding to the N‐terminal MB2 domain but still allows SKP2 to act as a C‐MYC cofactor by binding to the C‐terminal bHLH‐LZ domain, thereby endowing C‐MYC with sufficient capacity to initiate the transcription of a panel of oncogenic targets including E2Fs, MCMs, Cyclins and SKP2 (Figures 2 and 5C–E). Unlike the tumor suppressor ARF, which also stabilizes C‐MYC by antagonizing SKP2 but induces EGR1‐dependent apoptosis,^[^ ^18^ ^]^ PDCD11 does not activate the C‐MYC‐EGR1 axis (Figure S4F, Supporting Information), indicating that PDCD11 acts as a tumor‐promoter rather than an apoptosis‐inducer in p53‐mutant cancer cells.

As C‐MYC overexpression and p53 mutations in cancer cells cooperate to induce highly aggressive phenotypes and poor responses to treatment, C‐MYC has become an appealing therapeutic target for developing anticancer drugs especially when p53 is mutated.^[^ ^4^, ^33^ ^]^ However, the intrinsically disordered functional domains of C‐MYC and the lack of enzymatically active pockets limit the efficacy of traditional approaches that directly target this oncoprotein. To overcome this, indirect targeting strategies that affect C‐MYC stability have recently been highlighted, owing to their potential clinical value.^[^ ^5^ ^]^ A panel of DUB‐targeted small molecule drugs such as AZ1‐4, Vismodegib, P5091, FT827, and XL188 have been developed to degrade C‐MYC by unshackling the E3 ligase components, including FBXW7, FBXL14, and TRIM32.^[^ ^34^ ^]^ Considering the oncogenic activity of SKP2 by ubiquitinating p27 or other tumor suppressors or enhancing C‐MYC‐regulated transcription,^[^ ^35^ ^]^ in the current opinion, it is not a brilliant choice to directly activate SKP2 for C‐MYC degradation. Our findings provide a PDCD11‐targeted therapeutic rationale for specially degrading the C‐MYC oncoprotein by efficiently harnessing the SCF^SKP2^‐possessed E3 ubiquitination activity, which results in a reduced SKP2 expression to avoid triggering SKP2‐promoted tumor progression (Figures 2, 3, and 5). To probe this, our future work will include the development of RNAi agents^[^ ^36^ ^]^ against PDCD11 or antibodies/peptides/small molecules antagonizing PDCD11‐C‐MYC binding, which may exert promising efficacy by abrogating MYC oncogenic function in p53‐mutant malignancies.

More than a partner of C‐MYC oncoprotein in p53‐mutant breast and colon cancers, our previous^[^ ^11^ ^]^ and present findings conclude that PDCD11 probably drives progression of a broad range of malignancies no matter p53 is wild‐typed or mutated (Figure S1, Supporting Information). By forming a PDCD11‐p53‐HDM2 complex through direct interaction, PDCD11 destroys p53 to accelerate the growth of p53‐WT tumors, but switches to coordinate with C‐MYC to trigger oncogenic signaling when p53 is mutated. Unlike in p53‐mutant cancer cells, PDCD11 barely affect the C‐MYC level in p53‐WT cells, which might be attributed to the high level of HDM2 induced by WT p53^[^ ^11^ ^]^ and the likely overlapping binding sites of PDCD11 (1033–1431) with HDM2^[^ ^11^ ^]^ and C‐MYC (Figure 4; Figures S2C and S6, Supporting Information). Most likely, HDM2 occupies the C‐MYC‐binding domain on PDCD11 to deactivate the PDCD11‐C‐MYC axis and instead facilitate the degradation of the p53 tumor suppressor.^[^ ^11^ ^]^ Upon this hypothesis is solidly confirmed in the future, the undeveloped PDCD11 antagonists would be promising therapeutic drugs against a broad spectrum of human cancers by hindering the formation of either the PDCD11‐HDM2 or PDCD11‐C‐MYC complex.

## 4 Conclusion

Thus, “extra‐nucleolar” PDCD11 is an interacting partner of the C‐MYC oncoprotein in the nucleoplasm of p53‐mutant breast and colon cancer cells, which acts as a biomarker for aggressive tumor progression and grave prognosis. By occupying MB2 but not the bHLH‐LZ domain of C‐MYC, PDCD11 competitively hinders SKP2‐C‐MYC binding, thereby stabilizing C‐MYC to hyperactivate downstream oncogenic signaling. PDCD11 silencing restores SKP2‐mediated ubiquitination‐proteasomal degradation of C‐MYC to suppress tumor growth and metastasis, thus highlighting a potential PDCD11‐targeted regimen to combat p53‐mutant malignancies by indirectly destroying “undruggable” C‐MYC.

## 5 Experimental Section

### Key Resources

All the key resources used in this study were shown in Table S2 (Supporting Information). Sequences of shRNA/siRNA oligonucleotides and the primers used for qRT‐PCR were listed in Table S3 (Supporting Information).

### Cell Culture

MDA‐MB‐231*^SKP2‐/‐^* cells were constructed by Ubigene Biosciences Co., Ltd. (Guangzhou, China). sgRNA (Table S3, Supporting Information) was used to guide Cas9‐mediated SKP2 knockout, which was verified by genomic sanger sequencing. Source of the other cell lines was shown in Table S2 (Supporting Information). MDA‐MB‐231*^SKP2+/+^*, MDA‐MB‐231*^SKP2−/−^*, and Lenti‐X 293T cells were cultured in DMEM medium supplemented with 10% fetal bovine serum (FBS), 2 mm L‐glutamine, 100 U mL^−1^ penicillin, and 100 mg mL^−1^ streptomycin. HT‐29 and HCT116 cells were cultured in McCoy's 5A medium with the same supplements. All the cells were cultured at 37 °C in a humidified incubator with a 5% CO_2_ atmosphere. To ensure the reliability of data, all the cell experiments were completed within a cell passage number no more than 10.

### Gene Knockdown and Overexpression

All the lentiviral‐related plasmids were shown in Table S2 (Supporting Information). Lentivirus packaging and transduction were performed according to the previous work.^[^ ^11^, ^37^ ^]^ Forty eight hours after transduction, cells were selected with 2 µg mL^−1^ puromycin (for pLKO/pLVX‐Puro‐derived lentiviruses) or 10–30 µg mL^−1^ blasticidin S (for pLenti6‐derived lentivirus) to establish stable cell strains including MDA‐MB‐231 and HT‐29 cells coexpressing V5‐C‐MYC and HA‐SKP2, and MDA‐MB‐231, HT‐29, and HCT116 (previously constructed)^[^ ^11^ ^]^ cells inducibly expression shLuc, shPDCD11#1, or shPDCD11#2. A 100 ng mL^−1^ Doxy was used to induce PDCD11 silencing in the cells. The pcDNA3 plasmids or siRNAs (Tables S2 and S3, Supporting Information) were transfected into the cells to achieve transient gene overexpression or knockdown.

### In Vivo Antitumor Assay

Female 5‐week‐old BALB/c*‐Foxn1^nu^* nude mice weighing 18–22 g were provided by the Experimental Animal Center of Yangzhou University (Yangzhou, Jiangsu, China). To construct in situ breast cancer models, 5 × 10^6^ MDA‐MB‐231 cells transduced with inducible shLuc/shPDCD11#1 lentiviruses were injected into the fat pad of the lower left breast. To construct subcutaneous tumor models, 5 × 10^6^ HT‐29 cells transduced with inducible shRNA lentiviruses were subcutaneously injected into the upper right axilla. When the tumors grew to ≈3 mm in diameter, the mice were fed with 2 mg mL^−1^ Doxy in drinking water containing 5% sucrose to induce PDCD11 silencing. Tumor volumes were measured every three days and the operators were blinded to the grouping information. The mice bearing MDA‐MB‐231 tumors were sacrificed after a 21‐day treatment course, whereas the HT‐29‐bearing mice were sacrificed after a 15‐day course. All the primary tumors were harvested to be photographed and then subjected to Ki67 immunohistochemical assay. The lungs of MDA‐MB‐231‐bearing mice were also harvested for H&E staining to assess the pulmonary metastasis originating from breast tumors. To assess the hematogenous metastasis of cancer cells, 10^6^ MDA‐MB‐231 cells transduced with inducible shRNA lentiviruses were intravenously injected. After treatment with Doxy for 35 days, the nude mice were sacrificed and the lungs were harvested for H&E staining analysis.

### Cell Growth and Cell Cycle

The cell growth curves were drawn by counting cells at different time points. Cell cycle distribution was analyzed by performing PI staining assay according to the previous work.^[^ ^11^, ^37^ ^]^

### Cell Migration

Cell migration was investigated by a transwell assay. MDA‐MB‐231 (5 × 10^4^) or HT‐29 (5 × 10^5^) cells were resuspended in 200 µL medium without FBS and then seeded to a transwell chamber (8.0 µm pore size) which was placed in a 24‐well plate containing 600 µL medium with 10% FBS. Twenty‐four hours later, the cells in the top chamber were removed, whereas the cells in the bottom chamber were fixed in 4% paraformaldehyde and stained by 1×Giemsa to be photographed under a microscope.

### RNA‐seq

After treated with doxy to induce the expression of shLuc or shPDCD11#1 (*n* = 3), lentiviral‐transduced MDA‐MB‐231 cells were harvested and sent to Majorbio Technology Co., Ltd. (Shanghai, China) for whole transcriptome sequencing and analysis. Raw data for this sequencing were available from the Gene Expression Omnibus (GSE275527).

### qRT‐PCR and Western Blot Analyses

qRT‐PCR and WB analyses were performed to assess the mRNA and protein levels of target genes according to the previous work.^[^ ^11^, ^30^, ^37^ ^]^ The primer and antibody information was shown in Tables S2 and S3 (Supporting Information).

### CHX Chase Assay

To evaluate the stability of C‐MYC protein, cells were treated with 50 µg mL^−1^ CHX to arrest mRNA translation to protein.^[^ ^7b^ ^]^ WB analyses were performed to quantify the remaining C‐MYC after different treatment times.

### Prediction for the Interactors with PDCD11

Candidate interactors with PDCD11 were screened from HitPredict database (http://www.hitpredict.org).^[^ ^24^ ^]^

### IF Assay

The IF assay using anti‐PDCD11 (homemade) and anti‐C‐MYC (E5Q6W) was performed to investigate the colocalization of PDCD11 and C‐MYC in the cells. The detailed process for this assay was shown in the previous publication.^[^ ^11^ ^]^

### IP‐Ubiquitination and co‐IP Assays

To assess the level of ubiquitinated C‐MYC, cells were treated with proteasome inhibitor MG132 to prevent degradation of ubiquitinated proteins.^[^ ^22^ ^]^ Anti‐C‐MYC (Cat# A19032, Abclonal) or anti‐V5 (3C8) was used to immunoprecipitate the endogenous C‐MYC or the transduced C‐MYC fusing with V5‐Tag. The ubiquitinated C‐MYC was detected by WB using anti‐Ubiquitin (Cat# A19686, Abclonal). To investigate the interactions between C‐MYC and other proteins, anti‐C‐MYC or anti‐V5 was also used to immunoprecipitate the complexes comprising C‐MYC/V5‐C‐MYC and their interactors, which were detected by WB using the corresponding antibodies, including anti‐PDCD11 (homemade), anti‐C‐MYC (E5Q6W), anti‐S‐Tag (D2K2V), anti‐HA (C29F4), anti‐Cullin1 (Cat# A19034, Abclonal). The HRP‐linked secondary antibody against rabbit IgG light chain was used to avoid the interference by the antibody heavy chain.

### GST‐Pulldown Assay

GST‐pulldown assay was performed to investigate the PDCD11‐C‐MYC interaction in vitro and identify the binding regions according to the previous work.^[^ ^11^, ^37^ ^]^ The coding genes of PDCD11 and its truncated variants were cloned into pET‐32a(+), whereas the coding genes of C‐MYC and its truncations were cloned into pGEX‐6P‐1. These plasmids were transformed into *E. coli* BL21(DE3) (for C‐MYC and its truncations) or Rosseta(DE3) (for PDCD11 and its truncations) for the IPTG‐induced expression of the recombinant proteins with His‐S or GST tags, which were purified by affinity chromatography. Glutathione Sepharose 4B beads were incubated with the purified GST‐tagged and His‐S‐tagged proteins in turn. After washing 4 times with 1% Triton X‐100 in PBS, the beads were boiled in SDS‐PAGE sample loading buffer for WB analyses using anti‐S‐Tag (Cat# AF0285, Beyotime) and anti‐GST (homemade).

### Prediction for the Structure of Protein‐Protein Complexes

ColabFold, an AlphaFold2‐based Google online tool,^[^ ^2^, ^25^ ^]^ was used to predict protein‐protein interactions.

### Transcription Factor Activity of C‐MYC

Firefly luciferase reporter gene assay was performed to determine the TFA of C‐MYC. The cells stably coexpressing V5‐C‐MYC and HA‐SKP2 were cotransfected with pMyc‐TA‐Luc,^[^ ^27^ ^]^ pcDNA3‐derived plasmids, and siNC/siSKP2. 48 h later, cells were harvested and 2 × 10^5^ cells from each group were lysed in 200 µL Cell Lysis Buffer, followed by the procedures per the manual of Enhanced Firefly Luciferase Reporter Gene Assay Kit II (Cat# 632 180, Beyotime).

### Statistical Analysis

To compare the PDCD11 transcriptional levels in normal tissues, p53‐mutant tumors, and p53‐WT tumors, UALCAN (https://ualcan.path.uab.edu/index.html)^[^ ^12^ ^]^ was used to analyze the data from TCGA database. For clinical survival analysis, Kaplan–Meier plotter (https://kmplot.com/analysis/)^[^ ^13^ ^]^ was used to analyze the correlation between the PDCD11 level and probability of OS (overall survival), RFS (recurrence‐free survival), or DMFS (distant metastasis‐free survival) in p53‐mutant breast (lymph node‐positive) and colon (Stage T1‐T3) cancer patients. To reveal the correlation between the PDCD11 level and transcription of G1/S‐related C‐MYC target genes in tumoral and pericarcinomatous tissues, Pearson correlation analysis was performed using GEPIA2 (http://gepia2.cancer‐pku.cn/)^[^ ^19^ ^]^ to analyze the data from TCGA database. All the other statistical analyses were performed using GraphPad Prism 9. All the numerical data are shown as mean ± SD. Significance of the difference between two groups was determined using two‐tailed Student's t‐test and the variance similar between the groups was statistically compared using F‐test, whereas one‐way ANOVA was used for the comparison of more than two groups. ^*^ *p <* 0.05; ^**^ *p <* 0.01; ^***^p < 0.001; ^****^p < 0.0001 denote significant difference; NS denotes no significance.

### Ethics Approval and Consent to Participate

All the animal experiments were approved by the Institutional Animal Care and Use Committee of Yangzhou University (Approval No. 202 402 057) and performed in compliance with the guidelines of Jiangsu Laboratory Animal Welfare and Ethical Committee of Jiangsu Administrative Committee of Laboratory Animals.

## Conflict of Interest

The authors declare no conflict of interest.

## Author Contributions

L.D. and W.N. contributed equally to this work. L.D. conceived, designed, carried out the whole study, and wrote the manuscript; W.N., Y.M., L.X., Z.Z., K.L., Jingwen.L., X.M., Z.W., H.G., and Jiajia.L. performed the experiments and analyzed the data. D.T. and X.Z. supervised this project, and reviewed the manuscript. All authors read and approved the final manuscript.

## Supporting information



Supporting Information

## Data Availability

The data that support the findings of this study are available from the corresponding author upon reasonable request.
