# Supplementary material for: PDCD11 Stabilizes C‐MYC Oncoprotein by Hindering C‐MYC‐SKP2 Negative Feedback Loop to Facilitate Progression of p53‐Mutant Breast and Colon Malignancies
Source: Adv Sci (Weinh). 2025 Mar 7;12(17):2502416. doi: 10.1002/advs.202502416 (PMC12061330; doi:10.1002/advs.202502416)
Supplement: Supplementary file 1 — Supporting Information [file ADVS-12-2502416-s001.docx]

Supporting Information

**PDCD11 stabilizes C-MYC oncoprotein by hindering C-MYC-SKP2 negative feedback loop to facilitate progression of p53-mutant breast and colon malignancies**

Li Ding*, Wei Ni, Yichao Ma, Lin Xu, Zhiping Zhang, Kai Liao, Jingwen Li, Xinyu Mei, Zhun Wang, Huiqian Ge, Jiajia Li, Dong Tang, Xinyue Zhang*

**Table of contents**

**Figure S1.** PDCD11 is positively expressed in a panel of cancer types with p53 mutation.

**Figure S2.** PDCD11 loses the capacity to regulate p53-R280K due to the low HDM2 level in MDA-MB-231 cells but shows a greatly enhanced affinity with p53-R273H to downregulate it in HT-29 cells.

**Figure S3.** SDS-PAGE analyses of purified recombinant proteins.

**Figure S4.** PDCD11 regulates G1/S-related pathways to facilitate tumor progression.

**Figure S5.** PDCD11 expression positively correlates with the transcriptional levels of G1/S-related C-MYC targets in breast and colon cancer cases.

**Figure S6.** Wild-type p53 induces a high level of HDM2 which is likely to complete with C-MYC to interact with PDCD11 and disenable PDCD11 to regulate C-MYC in HCT116 cells.

**Figure S7.** PDCD11 regulates C-MYC ubiquitination independently of the FBXW7 level.

**Figure S8.** SKP2 knockout in MDA-MB-231 cells was verified by genomic sanger sequencing.

**Table S1.** Predicted interactions for PDCD11.

**Table S2.** Key resources used in this study.

**Table S3.** Oligonucleotides in this study.


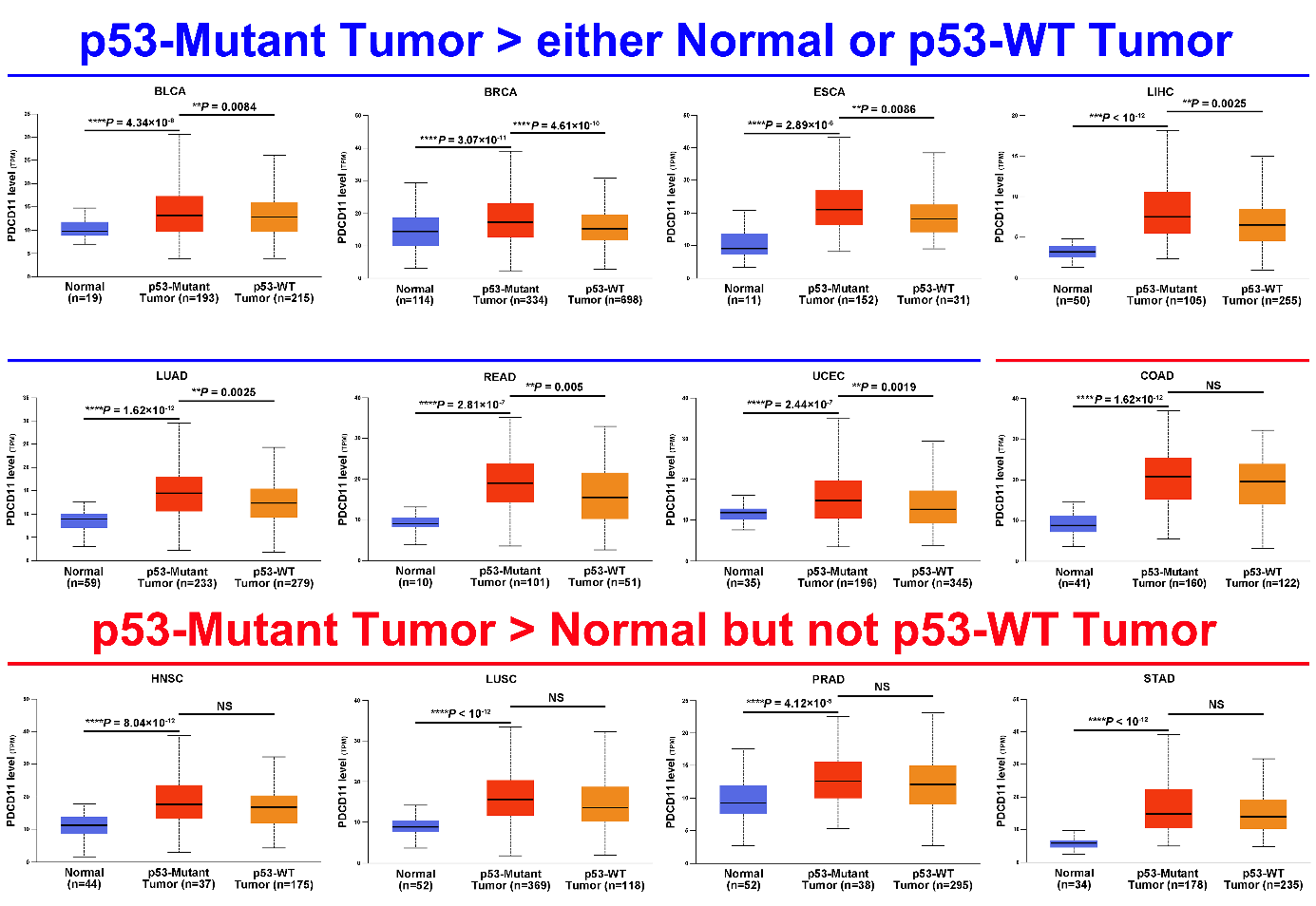


**Figure S1. PDCD11 is positively expressed in a panel of cancer types with p53 mutation.** PDCD11 transcriptional levels were compared in normal tissues, p53-mutant tumors, and p53-WT tumors by using UALCAN to analyzing the data from The Cancer Genome Atlas (TCGA) database. The cancer types which possess higher PDCD11 levels in p53-mutant tumors than in either p53-WT tumors or normal tissues were marked blue, whereas the other types possessing higher PDCD11 levels in p53-mutant tumors than in normal tissues but not p53-WT tumors were marked red. **P* < 0.05; ***P* < 0.01; ****P* < 0.001; *****P* < 0.0001 denote significant difference; NS denotes no significance.


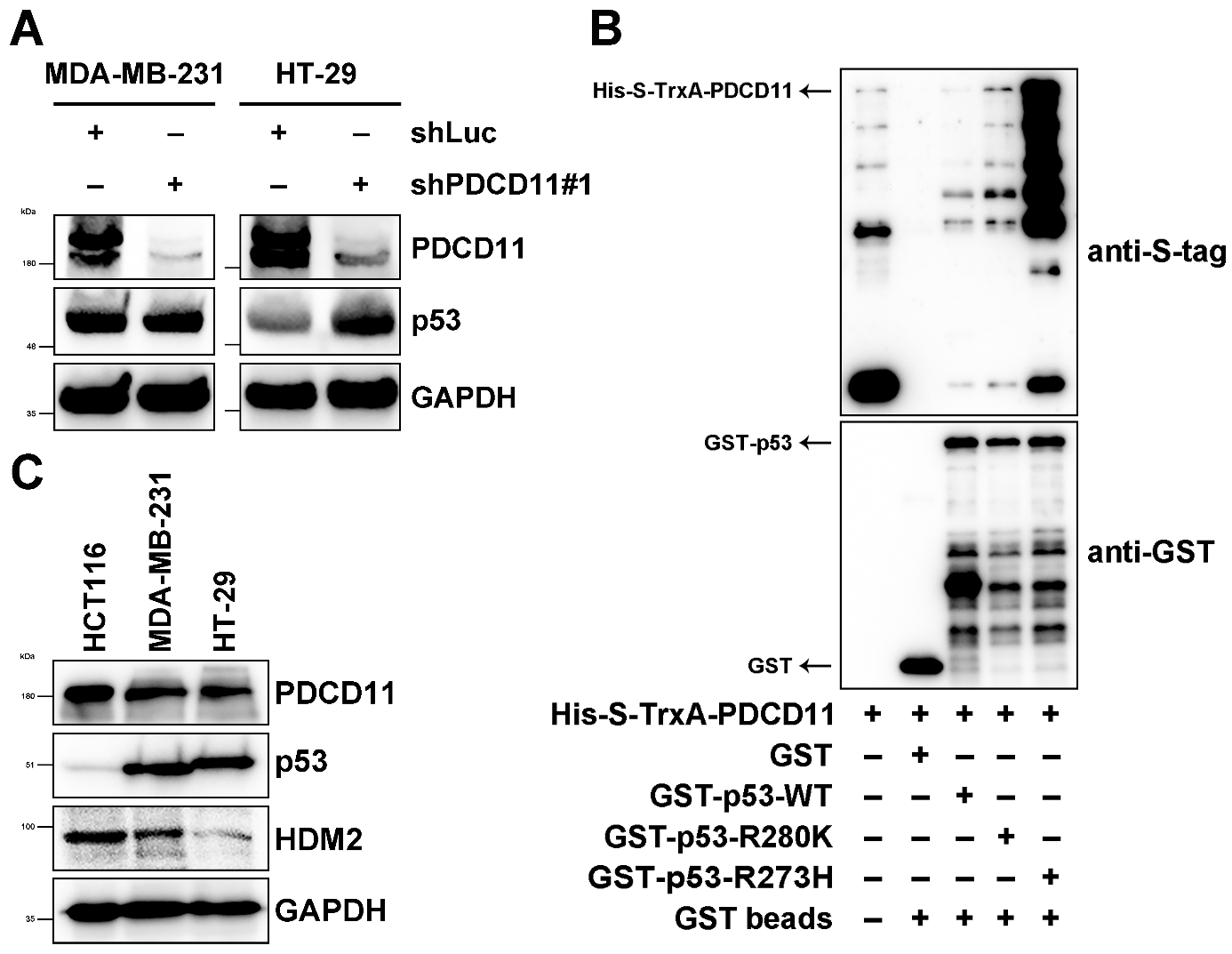


**Figure S2. PDCD11 loses the capacity to regulate p53-R280K due to the low HDM2 level in MDA-MB-231 cells but shows a greatly enhanced affinity with p53-R273H to downregulate it in HT-29 cells.** A) Lentivirus-transduced cells were treated with doxycycline (Doxy) to induce expression of shRNAs. Western blot (WB) analyses were performed to determine the levels of mutant p53 protein in MDA-MB-231 (p53-R280K) and HT-29 (p53-R273H) cells. GAPDH was used as loading control. B) GST-pulldown analyses were performed to investigate the interaction between PDCD11 and wild-type (WT)/mutant p53. C) WB analyses were performed to compare the levels of PDCD11, p53, and HDM2 proteins in HCT116, MDA-MB-231, and HT-29 cells with p53-WT, p53-R280K, and p53-R273H, respectively. GAPDH was used as loading control.


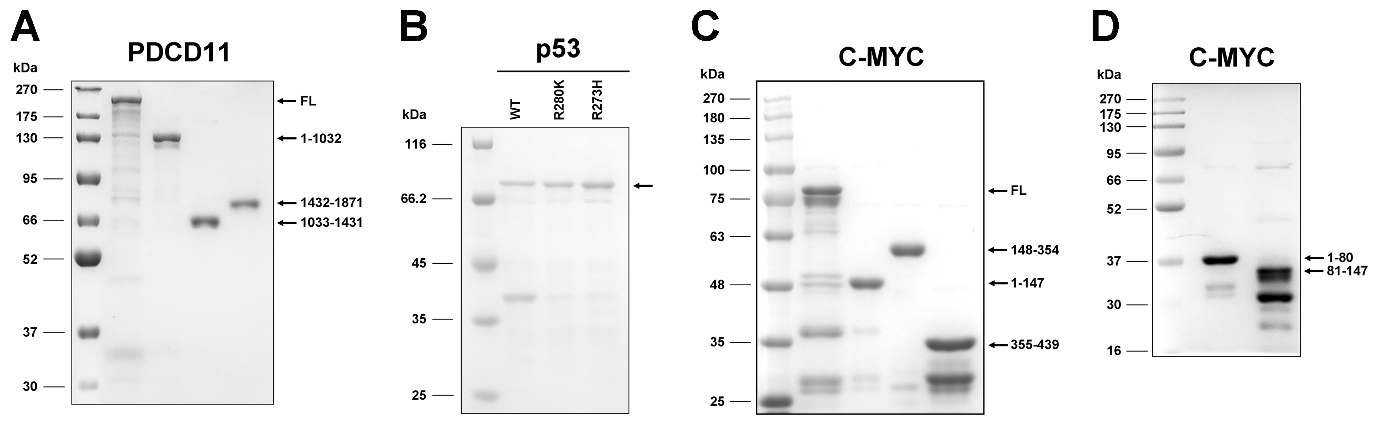


**Figure S3. SDS-PAGE analyses of purified recombinant proteins.** All the recombinant proteins were expressed in *Escherichia coli* and purified by affinity chromatography. A) His-S-tagged full-length PDCD11 (PDCD11-FL) and its truncations (aa 1-1032, aa 1033-1431, and aa 1432-1871). B) GST-tagged p53-WT, p53-R280K, and p53-R273H. C) GST-tagged C-MYC-FL and its truncations (aa 1-147, aa 148-354, and aa 355-439). D) GST-tagged C-MYC (1-80) and C-MYC (81-147).


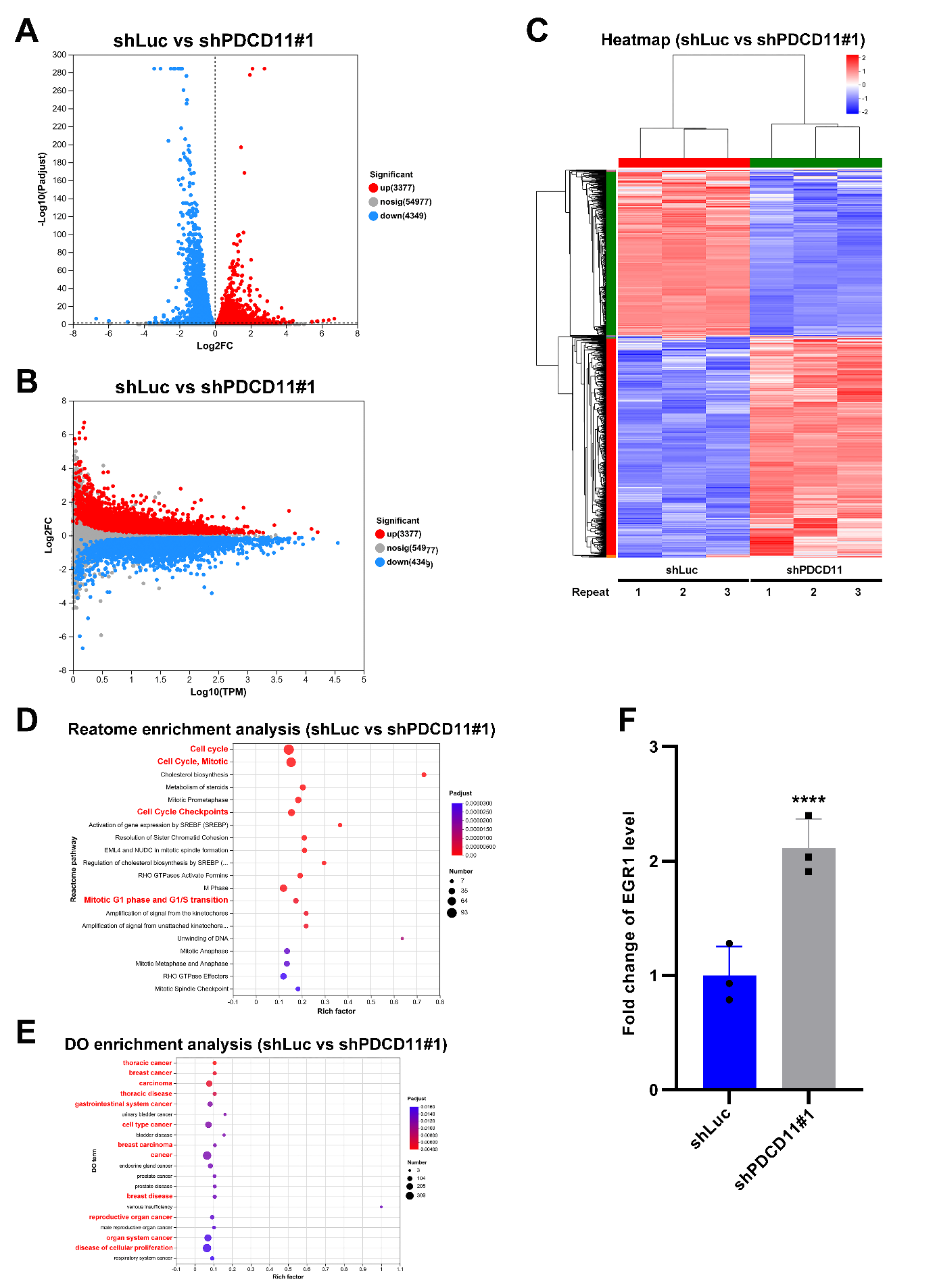


**Figure S4. PDCD11 regulates G1/S-related pathways to facilitate tumor progression.** Lentivirus-transduced MDA-MB-231 cells were treated with Doxy to induce expression of shRNAs and then subjected to RNA-seq analysis (n = 3). A and B) Volcano (A) and MA (B) plots were generated to summarize the number of differentially expressed genes (DEGs). C) A Heatmap showing the transcriptomic changes in response to PDCD11 silencing. D) Reactome enrichment analysis reveals the top 20 biological processes related to the DEGs. E) Disease Ontology (DO) enrichment analysis reveals the top 20 disease types related to the DEGs. F) Fold change of the transcriptional level of EGR1, a noncanonical C-MYC target. Data are shown as mean ± SD. **P* < 0.05; ***P* < 0.01; ****P* < 0.001; *****P* < 0.0001 denote significant difference; NS denotes no significance.


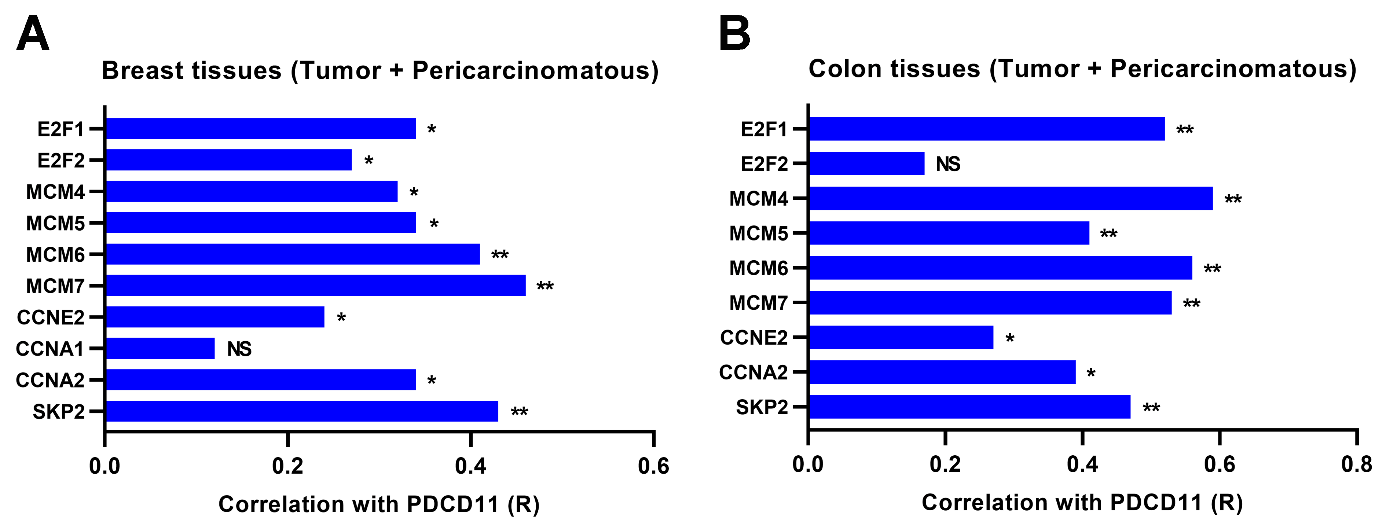


**Figure S5. PDCD11 expression positively correlates with the transcriptional levels of G1/S-related C-MYC targets in breast and colon cancer cases.** A and B) TCGA-based Pearson analyses was performed using GEPIA2 to investigate the correlation between the PDCD11 level and transcription of G1/S-related C-MYC target genes in tumoral and pericarcinomatous tissues. The raw data were obtained from TCGA databases. R > 0 indicates positive correlation; R < 0 indicates negative correlation; |R| = 0 ~ 0.2 denotes very weakly or not correlated (NS); |R| = 0.2 ~ 0.4 denotes weakly correlated (*); |R| = 0.4 ~ 0.6 denotes moderately correlated (**); |R| = 0.6 ~ 0.8 denotes strongly correlated (***); |R| = 0.8 ~ 1 denotes extremely strongly correlated (****).


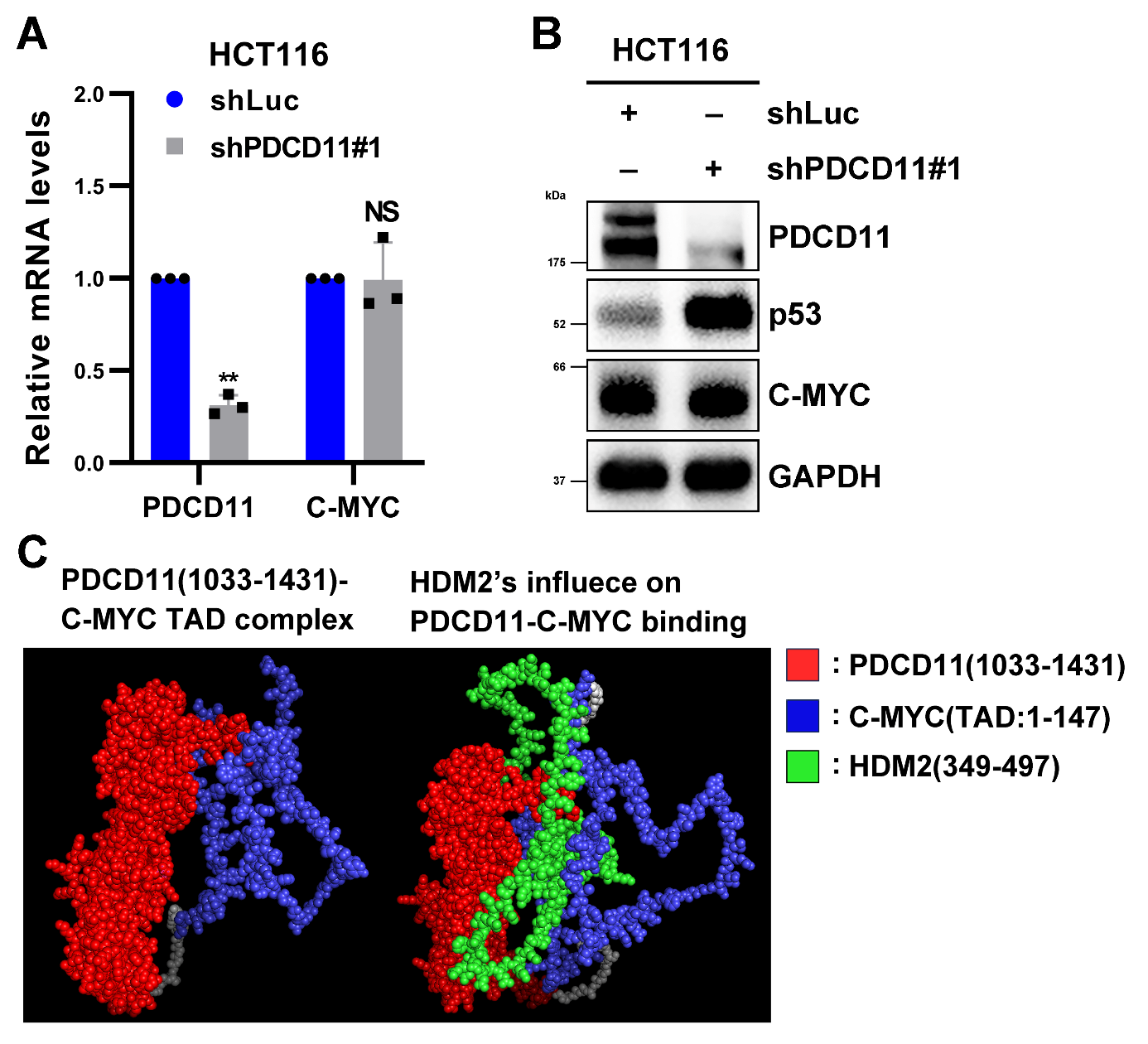


**Figure S6. Wild-type p53 induces a high level of HDM2 which is likely to complete with C-MYC to interact with PDCD11 and disenable PDCD11 to regulate C-MYC in HCT116 cells.** A and B) Lentivirus-transduced HCT116 cells were treated with Doxy to induce expression of shRNAs. A) Relative mRNA levels of PDCD11 and C-MYC were assessed by qRT-PCR (n = 3). GAPDH was used as internal control to normalize the values. The normalized values of control cells were set to 1. Data are shown as mean ± SD. **P* < 0.05; ***P* < 0.01; ****P* < 0.001; *****P* < 0.0001 denote significant difference; NS denotes no significance. B) The levels of PDCD11, p53, and C-MYC proteins were assessed by western blotting and GAPDH was used as loading control. C) ColabFold was used to predict the structure of PDCD11 (1033-1431)-C-MYC (1-147) complex and the influence of HDM2 (349-497) on PDCD11-C-MYC interaction.


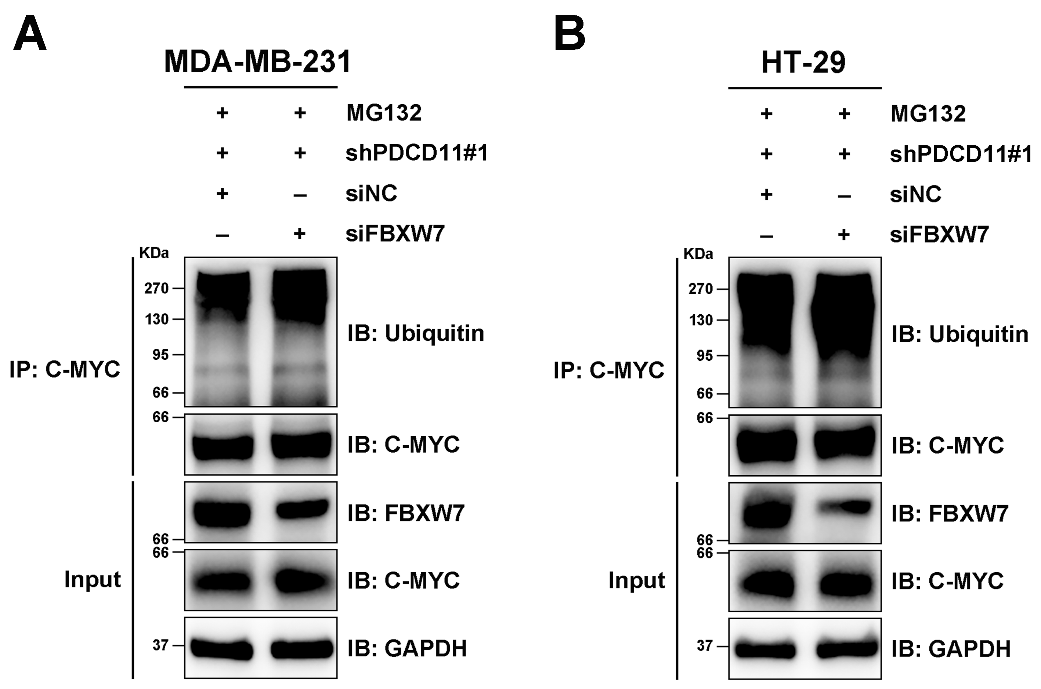


**Figure S7. PDCD11 regulates C-MYC ubiquitination independently of the FBXW7 level.** A and B) Lentivirus-transduced cells were treated with Doxy to induce expression of shPDCD11, followed by transfection of siNC/siFBXW7. Ubiquitinated and non-ubiquitinated C-MYC was immunoprecipitated using anti-C-MYC from the cells treated with MG132 (20 µM, 8 h). Anti-Ubiquitin and anti-C-MYC were used to detect ubiquitinated and non-ubiquitinated C-MYC, respectively. The levels of PDCD11, C-MYC, and FBXW7 proteins in the input cell lysate were determined by western blotting. GAPDH was used as loading control.


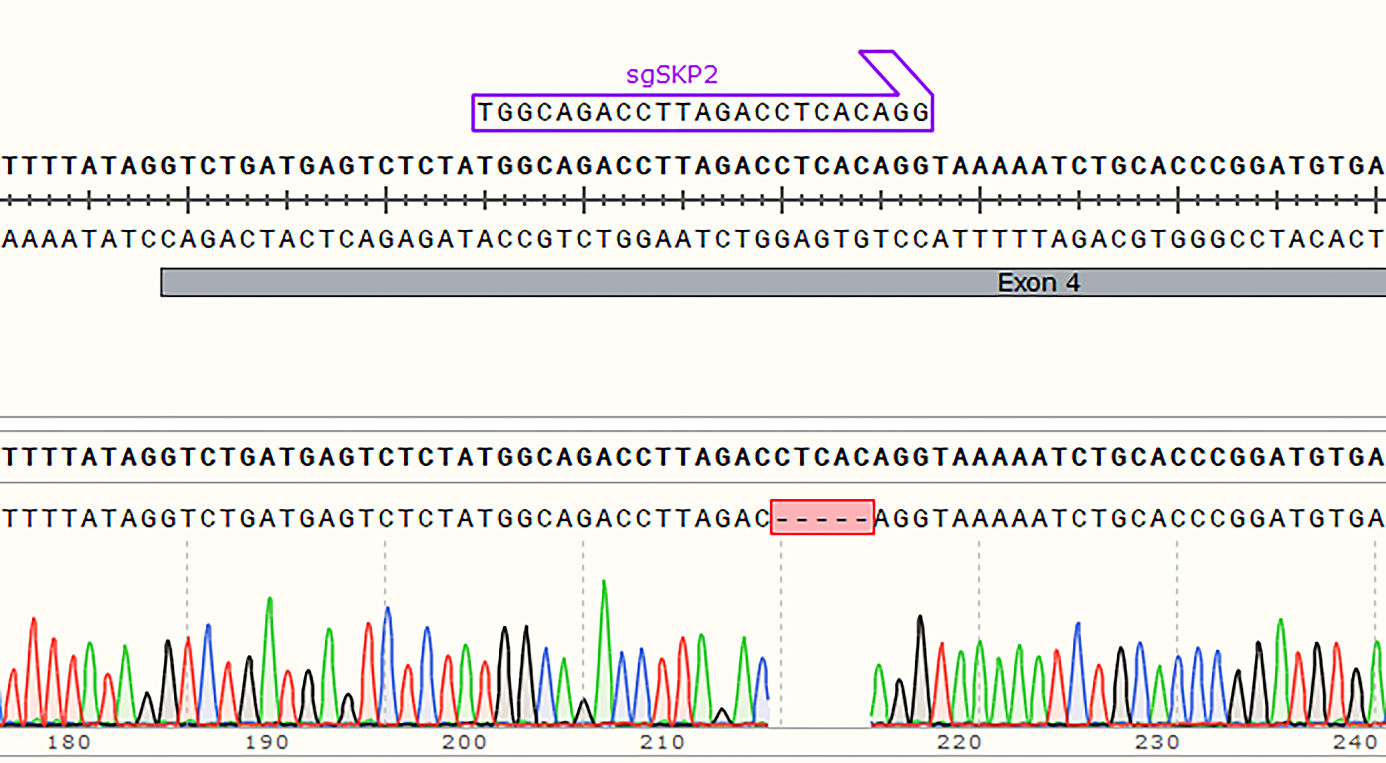


**Figure S8. SKP2 knockout in MDA-MB-231 cells was verified by genomic sanger sequencing.** sgSKP2 guides Cas9 protein to cleave the target gene, leading to a homozygous frameshift mutation of *SKP2* (5-bp deletion on Exon 4).

**Table S1. Predicted interactions for PDCD11.** The below data were queried from HitPredict database (http://www.hitpredict.org/index.html).

| **Interactor Name** | **Experiment Number** | **Category** | **Interaction Score** | **Confidence** |
| --- | --- | --- | --- | --- |
| SRPK2 | 2 | High-throughput | 0.811 | High |
| SRPK3 | 2 | High-throughput | 0.811 | High |
| **MYC** | **5** | **High-throughput** | **0.702** | **High** |
| KPSH1 | 1 | High-throughput | 0.691 | High |
| E2AK2 | 1 | High-throughput | 0.691 | High |
| CLK3 | 1 | High-throughput | 0.691 | High |
| GSK3B | 1 | High-throughput | 0.691 | High |
| RS14 | 1 | High-throughput | 0.691 | High |
| VRK3 | 1 | High-throughput | 0.691 | High |
| PDK1L | 1 | High-throughput | 0.691 | High |
| CDKL2 | 1 | High-throughput | 0.691 | High |
| SRPK1 | 1 | High-throughput | 0.691 | High |
| M3K14 | 1 | High-throughput | 0.691 | High |
| EXOS4 | 1 | High-throughput | 0.691 | High |
| NPM | 3 | High-throughput | 0.675 | High |
| RS6 | 3 | High-throughput | 0.656 | High |
| IF16 | 3 | High-throughput | 0.656 | High |
| FBRL | 3 | High-throughput | 0.64 | High |
| PKN2 | 1 | High-throughput | 0.631 | High |
| RRP8 | 2 | High-throughput | 0.628 | High |
| MECP2 | 2 | High-throughput | 0.628 | High |
| RL18A | 2 | High-throughput | 0.628 | High |
| RL18 | 2 | High-throughput | 0.628 | High |
| PKHO1 | 2 | High-throughput | 0.628 | High |
| ZN512 | 2 | High-throughput | 0.628 | High |
| RBM4 | 2 | High-throughput | 0.628 | High |
| H2AW | 2 | High-throughput | 0.628 | High |
| MAST1 | 1 | High-throughput | 0.618 | High |
| CTRO | 1 | High-throughput | 0.618 | High |
| RAF1 | 1 | High-throughput | 0.618 | High |
| CDK9 | 1 | High-throughput | 0.618 | High |
| HNRPK | 1 | High-throughput | 0.618 | High |
| RL26L | 1 | High-throughput | 0.618 | High |
| RL13A | 2 | High-throughput | 0.611 | High |
| RRP7A | 2 | High-throughput | 0.611 | High |
| LMNA | 2 | High-throughput | 0.601 | High |
| LN28A | 2 | High-throughput | 0.601 | High |
| SOX2 | 2 | High-throughput | 0.584 | High |
| GRSF1 | 2 | High-throughput | 0.584 | High |
| ANLN | 2 | High-throughput | 0.584 | High |
| ERBB2 | 2 | High-throughput | 0.576 | High |
| FGF3 | 2 | High-throughput | 0.574 | High |
| FGF8 | 2 | High-throughput | 0.574 | High |
| ESR1 | 2 | High-throughput | 0.571 | High |
| HNRC2 | 1 | High-throughput | 0.536 | High |
| MAGB2 | 1 | High-throughput | 0.536 | High |
| E2F6 | 1 | High-throughput | 0.536 | High |
| PRKRA | 1 | High-throughput | 0.536 | High |
| SURF6 | 1 | High-throughput | 0.536 | High |
| RLA0 | 1 | High-throughput | 0.536 | High |
| H14 | 1 | High-throughput | 0.536 | High |
| FOSL1 | 1 | High-throughput | 0.536 | High |
| H12 | 1 | High-throughput | 0.536 | High |
| RL17 | 1 | High-throughput | 0.536 | High |
| RS3 | 1 | High-throughput | 0.536 | High |
| RL13 | 1 | High-throughput | 0.536 | High |
| RL4 | 1 | High-throughput | 0.536 | High |
| MNDA | 1 | High-throughput | 0.536 | High |
| FRK | 1 | High-throughput | 0.536 | High |
| MAGA9 | 1 | High-throughput | 0.536 | High |
| KSYK | 1 | High-throughput | 0.536 | High |
| NOP2 | 1 | High-throughput | 0.536 | High |
| BAG6 | 1 | High-throughput | 0.536 | High |
| RL5 | 1 | High-throughput | 0.536 | High |
| RL28 | 1 | High-throughput | 0.536 | High |
| IMA5 | 1 | High-throughput | 0.536 | High |
| RL15 | 1 | High-throughput | 0.536 | High |
| RS16 | 1 | High-throughput | 0.536 | High |
| RL23A | 1 | High-throughput | 0.536 | High |
| RL8 | 1 | High-throughput | 0.536 | High |
| RED1 | 1 | High-throughput | 0.536 | High |
| HNRPU | 1 | High-throughput | 0.536 | High |
| CEBPZ | 1 | High-throughput | 0.536 | High |
| ILF3 | 1 | High-throughput | 0.536 | High |
| SRSF6 | 1 | High-throughput | 0.536 | High |
| G3BP1 | 1 | High-throughput | 0.536 | High |
| DGKZ | 1 | High-throughput | 0.536 | High |
| CUL3 | 1 | High-throughput | 0.536 | High |
| BMS1 | 1 | High-throughput | 0.536 | High |
| RRS1 | 1 | High-throughput | 0.536 | High |
| TEBP | 1 | High-throughput | 0.536 | High |
| PUM3 | 1 | High-throughput | 0.536 | High |
| SPT2 | 1 | High-throughput | 0.536 | High |
| TM10B | 1 | High-throughput | 0.536 | High |
| ZCCHV | 1 | High-throughput | 0.536 | High |
| NOP9 | 1 | High-throughput | 0.536 | High |
| LYRIC | 1 | High-throughput | 0.536 | High |
| CAND1 | 1 | High-throughput | 0.536 | High |
| ZC3H3 | 1 | High-throughput | 0.536 | High |
| SPB1 | 1 | High-throughput | 0.536 | High |
| RP25L | 1 | High-throughput | 0.536 | High |
| DDX54 | 1 | High-throughput | 0.536 | High |
| BRX1 | 1 | High-throughput | 0.536 | High |
| AEN | 1 | High-throughput | 0.536 | High |
| NOC3L | 1 | High-throughput | 0.536 | High |
| FGF13 | 1 | High-throughput | 0.536 | High |
| HDA11 | 1 | High-throughput | 0.536 | High |
| DDX27 | 1 | High-throughput | 0.536 | High |
| ZNF48 | 1 | High-throughput | 0.536 | High |
| RBM4B | 1 | High-throughput | 0.536 | High |
| BUD13 | 1 | High-throughput | 0.536 | High |
| RPP25 | 1 | High-throughput | 0.536 | High |
| MAK16 | 1 | High-throughput | 0.536 | High |
| SENP3 | 1 | High-throughput | 0.536 | High |
| NOL6 | 1 | High-throughput | 0.536 | High |
| RPF2 | 1 | High-throughput | 0.536 | High |
| DDX31 | 1 | High-throughput | 0.536 | High |
| I20L2 | 1 | High-throughput | 0.536 | High |
| DDX21 | 1 | High-throughput | 0.536 | High |
| RBM28 | 1 | High-throughput | 0.536 | High |
| ZSC32 | 1 | High-throughput | 0.536 | High |
| SRP68 | 1 | High-throughput | 0.536 | High |
| HERC5 | 1 | High-throughput | 0.536 | High |
| PURG | 1 | High-throughput | 0.536 | High |
| ABT1 | 1 | High-throughput | 0.536 | High |
| NOC2L | 1 | High-throughput | 0.536 | High |
| RBM19 | 1 | High-throughput | 0.536 | High |
| S10A8 | 1 | High-throughput | 0.518 | High |
| SYEP | 1 | High-throughput | 0.515 | High |
| NDC80 | 1 | High-throughput | 0.513 | High |
| POP7 | 1 | High-throughput | 0.513 | High |
| RPP40 | 1 | High-throughput | 0.513 | High |
| RPP29 | 1 | High-throughput | 0.513 | High |
| FANCC | 1 | High-throughput | 0.513 | High |
| TAF1A | 1 | High-throughput | 0.513 | High |
| AR13B | 1 | High-throughput | 0.513 | High |
| RSBN1 | 1 | High-throughput | 0.513 | High |
| NEPRO | 1 | High-throughput | 0.513 | High |
| CC137 | 1 | High-throughput | 0.513 | High |
| ZN467 | 1 | High-throughput | 0.513 | High |
| RM52 | 1 | High-throughput | 0.513 | High |
| GATD1 | 1 | High-throughput | 0.513 | High |
| XPF | 1 | High-throughput | 0.513 | High |
| NEIL1 | 1 | High-throughput | 0.513 | High |
| ZC3HA | 1 | High-throughput | 0.513 | High |
| CEP19 | 1 | High-throughput | 0.513 | High |
| LLPH | 1 | High-throughput | 0.513 | High |
| SSBP4 | 1 | High-throughput | 0.513 | High |
| TOLIP | 1 | High-throughput | 0.513 | High |
| CBX8 | 1 | High-throughput | 0.513 | High |
| ABC3C | 1 | High-throughput | 0.513 | High |
| CARF | 1 | High-throughput | 0.513 | High |
| TF3C4 | 1 | High-throughput | 0.513 | High |
| RL36 | 1 | High-throughput | 0.513 | High |
| NFKB1 | 1 | Small-scale | 0.506 | High |
| KLF4 | 1 | High-throughput | 0.5 | High |
| HMGN2 | 1 | High-throughput | 0.493 | High |
| PPM1A | 1 | High-throughput | 0.493 | High |
| ZC3HD | 1 | High-throughput | 0.493 | High |
| UTP16 | 1 | High-throughput | 0.493 | High |
| CD6 | 1 | High-throughput | 0.489 | High |
| JIP2 | 1 | High-throughput | 0.489 | High |
| SCRB2 | 1 | High-throughput | 0.489 | High |
| PNM8A | 1 | High-throughput | 0.489 | High |
| ZSC31 | 1 | High-throughput | 0.489 | High |
| WIF1 | 1 | High-throughput | 0.489 | High |
| MEN1 | 1 | High-throughput | 0.479 | High |
| NOP56 | 1 | High-throughput | 0.479 | High |
| RPA34 | 1 | High-throughput | 0.479 | High |
| PRC1 | 1 | High-throughput | 0.479 | High |
| SOX15 | 1 | High-throughput | 0.479 | High |
| OBSL1 | 1 | High-throughput | 0.479 | High |
| DNJB6 | 1 | High-throughput | 0.479 | High |
| EED | 1 | High-throughput | 0.479 | High |
| STAU1 | 1 | High-throughput | 0.479 | High |
| MYCN | 1 | High-throughput | 0.479 | High |
| P53 | 1 | High-throughput | 0.479 | High |
| H2A1B | 1 | High-throughput | 0.479 | High |
| ROA1 | 1 | High-throughput | 0.479 | High |
| PARP1 | 1 | High-throughput | 0.479 | High |
| H2AB2 | 1 | High-throughput | 0.479 | High |
| BRAF | 1 | High-throughput | 0.479 | High |
| RFA2 | 1 | High-throughput | 0.479 | High |
| H15 | 1 | High-throughput | 0.479 | High |
| NUCL | 1 | High-throughput | 0.479 | High |
| RFA1 | 1 | High-throughput | 0.479 | High |
| STIP1 | 1 | High-throughput | 0.479 | High |
| BMI1 | 1 | High-throughput | 0.479 | High |
| RFA3 | 1 | High-throughput | 0.479 | High |
| PP1G | 1 | High-throughput | 0.479 | High |
| COIL | 1 | High-throughput | 0.479 | High |
| RL3 | 1 | High-throughput | 0.479 | High |
| CCNF | 1 | High-throughput | 0.479 | High |
| ETV3 | 1 | High-throughput | 0.479 | High |
| RECQ1 | 1 | High-throughput | 0.479 | High |
| LHX1 | 1 | High-throughput | 0.479 | High |
| NR2C2 | 1 | High-throughput | 0.479 | High |
| CENPA | 1 | High-throughput | 0.479 | High |
| LHX2 | 1 | High-throughput | 0.479 | High |
| RAB9A | 1 | High-throughput | 0.479 | High |
| IF6 | 1 | High-throughput | 0.479 | High |
| WDR5 | 1 | High-throughput | 0.479 | High |
| UBE2H | 1 | High-throughput | 0.479 | High |
| H4 | 1 | High-throughput | 0.479 | High |
| RS24 | 1 | High-throughput | 0.479 | High |
| RL31 | 1 | High-throughput | 0.479 | High |
| KIF23 | 1 | High-throughput | 0.479 | High |
| TF65 | 1 | Small-scale | 0.479 | High |
| CHD3 | 1 | High-throughput | 0.479 | High |
| TIF1B | 1 | High-throughput | 0.479 | High |
| FKBP5 | 1 | High-throughput | 0.479 | High |
| BIRC3 | 1 | High-throughput | 0.479 | High |
| KLF9 | 1 | High-throughput | 0.479 | High |
| SMC1A | 1 | High-throughput | 0.479 | High |
| CHD4 | 1 | High-throughput | 0.479 | High |
| CUL7 | 1 | High-throughput | 0.479 | High |
| HAPR1 | 1 | Small-scale | 0.479 | High |
| SUZ12 | 1 | High-throughput | 0.479 | High |
| TNIP1 | 1 | High-throughput | 0.479 | High |
| KIF14 | 1 | High-throughput | 0.479 | High |
| E2F4 | 1 | High-throughput | 0.479 | High |
| PDS5A | 1 | High-throughput | 0.479 | High |
| ANR55 | 1 | High-throughput | 0.479 | High |
| LARP7 | 1 | High-throughput | 0.479 | High |
| RC3H1 | 1 | High-throughput | 0.479 | High |
| FTM | 1 | High-throughput | 0.479 | High |
| PAXI1 | 1 | High-throughput | 0.479 | High |
| H32 | 1 | High-throughput | 0.479 | High |
| PARPT | 1 | High-throughput | 0.479 | High |
| ZCH18 | 1 | High-throughput | 0.479 | High |
| DHX40 | 1 | High-throughput | 0.479 | High |
| SMC5 | 1 | High-throughput | 0.479 | High |
| SLX4 | 1 | High-throughput | 0.479 | High |
| RN168 | 1 | Small-scale | 0.479 | High |
| STAG2 | 1 | High-throughput | 0.479 | High |
| ZBTB2 | 1 | High-throughput | 0.479 | High |
| NUP43 | 1 | High-throughput | 0.479 | High |
| DOT1L | 1 | High-throughput | 0.479 | High |
| DNJC9 | 1 | High-throughput | 0.479 | High |
| ESR2 | 1 | High-throughput | 0.479 | High |
| RL36L | 1 | High-throughput | 0.479 | High |
| DCPS | 1 | High-throughput | 0.479 | High |
| CHM4C | 1 | High-throughput | 0.479 | High |
| OPTN | 1 | High-throughput | 0.479 | High |
| SNR40 | 1 | High-throughput | 0.479 | High |
| DDRGK | 1 | High-throughput | 0.479 | High |
| CIC | 1 | High-throughput | 0.479 | High |
| RING2 | 1 | High-throughput | 0.479 | High |
| EBP2 | 1 | High-throughput | 0.479 | High |
| GRWD1 | 1 | High-throughput | 0.479 | High |
| DDX23 | 1 | High-throughput | 0.479 | High |
| MK67I | 1 | High-throughput | 0.479 | High |
| RPA49 | 1 | High-throughput | 0.479 | High |
| SLIRP | 1 | High-throughput | 0.479 | High |
| WWTR1 | 1 | High-throughput | 0.479 | High |
| XRN2 | 1 | High-throughput | 0.479 | High |
| CCDC8 | 1 | High-throughput | 0.479 | High |
| RC3H2 | 1 | High-throughput | 0.479 | High |
| BRD7 | 1 | High-throughput | 0.479 | High |
| SIR7 | 1 | High-throughput | 0.479 | High |
| NLE1 | 1 | High-throughput | 0.479 | High |
| AATF | 1 | High-throughput | 0.479 | High |
| DDX56 | 1 | High-throughput | 0.479 | High |
| FZR1 | 1 | High-throughput | 0.479 | High |
| SNX6 | 1 | High-throughput | 0.479 | High |
| CE164 | 1 | High-throughput | 0.479 | High |
| SMC3 | 1 | High-throughput | 0.479 | High |
| DDX52 | 1 | High-throughput | 0.479 | High |
| ZN330 | 1 | High-throughput | 0.479 | High |
| KLF12 | 1 | High-throughput | 0.479 | High |
| MAFB | 1 | High-throughput | 0.479 | High |
| LRC71 | 1 | High-throughput | 0.47 | High |
| TTC28 | 1 | High-throughput | 0.47 | High |
| LEG9 | 1 | High-throughput | 0.458 | High |
| TLX2 | 1 | High-throughput | 0.458 | High |
| APEX1 | 1 | High-throughput | 0.458 | High |
| RL14 | 1 | High-throughput | 0.458 | High |
| RHOA | 1 | High-throughput | 0.458 | High |
| RB11A | 1 | High-throughput | 0.458 | High |
| MTF1 | 1 | High-throughput | 0.458 | High |
| DCAF4 | 1 | High-throughput | 0.458 | High |
| CHCH1 | 1 | High-throughput | 0.458 | High |
| RT26 | 1 | High-throughput | 0.458 | High |
| AKIP | 1 | High-throughput | 0.458 | High |
| UBP36 | 1 | High-throughput | 0.458 | High |
| SCC4 | 1 | High-throughput | 0.458 | High |
| JUN | 1 | High-throughput | 0.445 | High |
| RRP1B | 1 | High-throughput | 0.445 | High |
| NTRK1 | 1 | High-throughput | 0.437 | High |
| TRI67 | 1 | High-throughput | 0.437 | High |
| TT21A | 1 | High-throughput | 0.437 | High |
| LGR4 | 1 | High-throughput | 0.437 | High |
| ACE2 | 1 | High-throughput | 0.437 | High |
| PKHA4 | 1 | High-throughput | 0.437 | High |
| HECD1 | 1 | High-throughput | 0.437 | High |
| XPC | 1 | High-throughput | 0.426 | High |
| ARK74 | 1 | High-throughput | 0.426 | High |
| IFN14 | 1 | High-throughput | 0.279 | Low |
| KALM | 1 | High-throughput | 0.279 | Low |
| NOGG | 1 | High-throughput | 0.279 | Low |
| FGFP1 | 1 | High-throughput | 0.279 | Low |
| AKA28 | 1 | High-throughput | 0.279 | Low |
| USE1 | 1 | High-throughput | 0.279 | Low |
| PAI2B | 1 | High-throughput | 0.279 | Low |
| SGPL1 | 1 | High-throughput | 0.269 | Low |
| BET1 | 1 | High-throughput | 0.249 | Low |
| AT1A1 | 1 | High-throughput | 0.249 | Low |
| COX8A | 1 | High-throughput | 0.249 | Low |
| AUHM | 1 | High-throughput | 0.249 | Low |
| GRM2 | 1 | High-throughput | 0.249 | Low |
| MKS3 | 1 | High-throughput | 0.249 | Low |

**Table S2. Key resources used in this study.**

| REAGENT or RESOURCE | SOURCE | IDENTIFIER |
| --- | --- | --- |
| Antibodies |  |  |
| Anti-mouse IgG (H+L), HRP-linked  Anti-rabbit IgG (H+L), HRP-linked  Mouse monoclonal anti-V5 (3C8) | Solarbio  Solarbio  Solarbio | Cat# SE131  Cat# SE134  Cat# K200004M |
| Anti-rabbit IgG (LCS), HRP-linked  Anti-mouse IgG (H+L), Alexa Fluor 647-linked | Abbkine  Beyotime | Cat# A25022  Cat# A0473 |
| Anti-Rabbit IgG (H+L), FITC-linked  Mouse monoclonal anti-S-Tag | Beyotime  Beyotime | Cat# A0562  Cat# AF0285 |
| Mouse monoclonal anti-GAPDH  Mouse monoclonal anti-GST  Mouse monoclonal anti-PDCD11 | Homemade  Homemade  Homemade | N/A  N/A  DOI: 10.7150/ijms.3635 |
| Mouse monoclonal anti-p53 (DO-1) | Santa Cruz | Cat# sc-126; RRID: AB_628082 |
| Mouse monoclonal anti-p27 (F8) | Santa Cruz | Cat# sc-1641; RRID: AB_628074 |
| Mouse monoclonal anti-FLAG (M2) | Sigma-Aldrich | Cat# F1804; RRID: AB_262044 |
| Rabbit monoclonal anti-V5 (D3H8Q) | CST | Cat# 13202; RRID: AB_2687461 |
| Rabbit monoclonal anti-HA (C29F4)  Rabbit monoclonal anti-C-MYC (E5Q6W)  Rabbit monoclonal anti-S-Tag (D2K2V) | CST  CST  CST | Cat# 3724; RRID: AB_2924897  Cat# 18583; RRID: AB_2895543  Cat# 12774; RRID: AB_2798022 |
| Rabbit monoclonal anti-C-MYC | Abclonal | Cat# A19032; RRID: AB_2862524 |
| Rabbit monoclonal anti-E2F1  Rabbit monoclonal anti-CCNE2  Rabbit monoclonal anti-SKP2 | Abclonal  Abclonal  Abclonal | Cat# A19579; RRID: AB_2862679  Cat# A9305; RRID: AB_2863707  Cat# A4046; RRID: AB_2863176 |
| Rabbit monoclonal anti-Cullin1 | Abclonal | Cat# A19034; RRID: AB_2862526 |
| Rabbit monoclonal anti-Ubiquitin | Abclonal | Cat# A19686; RRID: AB_2862735 |
| Rabbit polyclonal anti-FBXW7 | Abclonal | Cat# A5872; RRID: AB_2766621 |
| Bacterial and Virus Strains |  |  |
| *E. coli.* Rosetta(DE3) Competent Cells  *E. coli.* BL21(DE3) Competent Cells  *E. coli.* Stbl3 Competent Cells | Beyotime  Thermo Fisher  Thermo Fisher | Cat# D1065  Cat# C600003  Cat# C737303 |
| *E. coli.* DH5α Competent Cells | Vazyme | Cat# C502 |
| Chemicals, Peptides, and Recombinant Proteins |  |  |
| RIPA Lysis Buffer  His-tag Purification Resin | Beyotime  Beyotime | Cat# P0013B  Cat# 2210 |
| Protease inhibitor cocktail | Beyotime | Cat# P1010 |
| Protein A+G magnetic beads | Beyotime | Cat# P2108 |
| Doxycycline | Beyotime | Cat# ST039 |
| Puromycin | Beyotime | Cat# ST551 |
| Antifade Mounting Medium | Beyotime | Cat# P0126 |
| MG132 | Beyotime | Cat# S1748 |
| Polybrene | Beyotime | Cat# C0351 |
| Lipo8000 | Beyotime | Cat# C0533 |
| Lipofectamine 2000 | Thermo Fisher | Cat# 11668019 |
| DMEM medium  Opti-MEM® I Reduced Serum Medium | Thermo Fisher  Thermo Fisher | Cat# 12430047  Cat# 31985062 |
| Trypsin-EDTA | Thermo Fisher | Cat# 25200072 |
| Blasticidin S | Solarbio | Cat# B9300 |
| Hoechst 33258 | Solarbio | Cat# B8030 |
| Total RNA Extraction Reagent | Vazyme | Cat# R401-01 |
| ECL Western Blotting Substrate | Vazyme | Cat# E412 |
| HiScript III RT SuperMix for qPCR | Vazyme | Cat# R323-01 |
| Fetal bovine serum | BIOODIN | Cat# UB68506 |
| Glutathione Sepharose 4B | GE Healthcare | Cat# 17-0756-01 |
| McCoy's 5A medium | Sigma-Aldrich | Cat# M4892 |
| Propidium iodide  IPTG | Sigma-Aldrich  Sigma-Aldrich | Cat# P4170  Cat# I6758 |
| Glutamine  Penicillin+Streptomycin  Ampicillin  Kanamycin  RNase A  *Age*Ⅰ RE  *Bam*HⅠ RE  *Eco*RⅠ RE  *Xho*Ⅰ RE  T4 DNA ligase | Sigma-Aldrich  Sigma-Aldrich  Sigma-Aldrich  Sigma-Aldrich  ABM  NEB  NEB  NEB  NEB  TransGen | Cat# 1294808  Cat# V900929  Cat# A9518  Cat# E004000  Cat# G117  Cat# R3552  Cat# R0136  Cat# R0101  Cat# R0146  Cat# FL101 |
| Critical Commercial Kits |  |  |
| HiScript III 1st Strand cDNA Synthesis Kit | Vazyme | Cat# R312 |
| BCA Protein Assay Kit | Vazyme | Cat# E112 |
| ClonExpress Ultra One Step Cloning Kit | Vazyme | Cat# C115-02 |
| Plasmid miniprep kit | Vazyme | Cat# DC201-01 |
| Gateway® LR Clonase® | Thermo Fisher | Cat# 11791043 |
| EndoFree Plasmid midiprep kit | TIANGEN | Cat# DP104 |
| Enhanced Firefly Luciferase Reporter Gene Assay Kit II | Beyotime | Cat# RG010 |
| Deposited data |  |  |
| RNA-seq: shLuc vs shPDCD11#1 | This study | GEO: GSE275527 |
| Experimental Models: Cell Lines |  |  |
| MDA-MB-231*^SKP2+/+^* | Cell Bank, CAS | TCHu227 |
| MDA-MB-231*^SKP2-/-^* | This study | N/A |
| HT-29  HCT116 | Cell Bank, CAS  Cell Bank, CAS | TCHu103  TCHu 99 |
| Lenti-X 293T | Clontech | Cat# 632180 |
| Experimental Models: Organisms/Strains |  |  |
| Mouse: BALB/c*-Foxn1^nu^* | MAC in YZU | SCXK(Su)2017-0007 |
| Oligonucleotides |  |  |
| Primers for qRT-PCR | This study | See Table S3 |
| shRNA/siRNA/sgRNA | This study | See Table S3 |
| Recombinant DNA |  |  |
| pCMV-VSV-G  pCMV-dR8.2  Tet-pLKO-Puro-shLuc  Tet-pLKO-Puro-shPDCD11#1 | Addgene  Addgene  Previous study  Previous study | Cat# 8454  Cat# 8455  DOI: 10.1038/s41389-023-00501-2  DOI: 10.1038/s41389-023-00501-2 |
| Tet-pLKO-Puro-shPDCD11#2 | This study | NA |
| pET-32a(+)-PDCD11 | Previous study | DOI: 10.1038/s41389-023-00501-2 |
| pET-32a(+)-PDCD11(1-1032) | Previous study | DOI: 10.1038/s41389-023-00501-2 |
| pET-32a(+)-PDCD11(1033-1431) | Previous study | DOI: 10.1038/s41389-023-00501-2 |
| pET-32a(+)-PDCD11(1432-1871)  pGEX-5X-1-p53-WT | Previous study  Previous study | DOI: 10.1038/s41389-023-00501-2  DOI: 10.1038/s41389-023-00501-2 |
| pGEX-6P-1  pGEX-6P-1-p53-R273H  pGEX-6P-1-p53-R280K | GE Healthcare  This study  This study | Cat. #28-9546-48  N/A  N/A |
| pGEX-6P-1-C-MYC | This study | N/A |
| pGEX-6P-1-C-MYC(1-147)  pGEX-6P-1-C-MYC(1-80)  pGEX-6P-1-C-MYC(81-147)  pGEX-6p-1-C-MYC(148-354)  pGEX-6p-1-C-MYC(355-439)  pcDNA3  pcDNA3-Luc  pcDNA3-C-MYC | This study  This study  This study  This study  This study  Thermo Fisher  Previous study  This study | N/A  N/A  N/A  N/A  N/A  Cat# V79020  DOI: 10.1038/s41389-023-00501-2  N/A |
| pcDNA3-PDCD11 | This study | N/A |
| pcDNA3-S-Tag-NLS-PDCD11(1033-1431) | This study | N/A |
| pLenti6-GW/V5-DEST | Thermo Fisher | Cat# V49610 |
| pLenti6-GW/V5-C-MYC | This study | N/A |
| pLVX-IRES-Puro  pLVX-HA-SKP2 | Takara  This study | Cat# 632164  N/A |
| pMyc-TA-luc | Beyotime | Cat# D2198 |
| Software and Algorithms |  |  |
| FlowJo V10 | FLOWJO, LLC | https://www.flowjo.com |
| ImageJ 1.52a | NIH | https://imagej.net/ij/ |
| Image-Pro Plus 6.0 | Meyer | https://www.meyerinst.com |
| LasX 3.3.0 | Leica | https://www.lasx.com/ |
| GraphPad Prism 9 | Dotmatics | https://www.graphpad.com |

**Table S3. Oligonucleotides in this study.**

| **Primers for qRT-PCR** | **Sequences (5’-3’)** |
| --- | --- |
| GAPDH_RT_Fwd | TGGGCTACACTGAGCACCAG |
| GAPDH_RT_Rev | GGGTGTCGCTGTTGAAGTCA |
| PDCD11_RT_Fwd | TTTGCCCAGCTTGAGTTTCAG |
| PDCD11_RT_Rev | TGTCGATATAGACCGACCAGACA |
| C-MYC_RT_Fwd | CGTCTCCACACATCAGCACAA |
| C-MYC_RT_Rev | TCTTGGCAGCAGGATAGTCCTT |
| E2F1_RT_Fwd | ACCCTGCAGAGCAGATGGTT |
| E2F1_RT_Rev | GGCTTGGAGCTGGGTCTCA |
| CCNE2_RT_Fwd | CCCAGCCAGACGGAATCC |
| CCNE2_RT_Rev | TTTTTTGACATCCTGGGTAGTTTTC |
| SKP2_RT_Fwd | AGAGGAGCCCGACAGTGAGA |
| SKP2_RT_Rev | GGGTGGCCCAGGTTTGAG |
| E2F2_RT_Fwd | GGCTGGCCTATGTGACTTACCA |
| E2F2_RT_Rev | GCAATCACTGTCTGCTCCTTAAAG |
| MCM2_RT_Fwd | TGTGATCGAAGACGACGTCAA |
| MCM2_RT_Rev | TGTGTCTATGAAGCTCTCCAGCAT |
| MCM4_RT_Fwd | AGGAAGGATTTTAGTCACACTGGAA |
| MCM4_RT_Rev | GTCGCCACACAGCAAGATGT |
| MCM5_RT_Fwd | CCATGGAGCAGCAGACCAT |
| MCM5_RT_Rev | GGAGCAGCGGGAGTTCAG |
| MCM6_RT_Fwd | GGCCGAGAAGTGCCAGAAA |
| MCM6_RT_Rev | TCCATCGCTGCTCTGAAACTC |
| MCM7_RT_Fwd | CCCACTTTCATGCCTCTGATC |
| MCM7_RT_Rev | CCTGAGCGGTTGGTTTGG |
| CCNA1_RT_Fwd | TTGGCCAGAAACCCTTGCT |
| CCNA1_RT_Rev | TCACTCAGGCAAGGCACAATT |
| CCNA2_RT_Fwd | ACGGGTTGCACCCCTTAAG |
| CCNA2_RT_Rev | CCAAGGAGGAACGGTGACA |
| **shRNA/siRNA/sgRNA** | **Sequences (5’-3’)** |
| Upper_shLuc | CCGGCGCTGAGTACTTCGAAATGTCCTCGAGGACATTTCGAAGTACTCAGCGTTTTT |
| Bottom_shLuc | AATTAAAAACGCTGAGTACTTCGAAATGTCCTCGAGGACATTTCGAAGTACTCAGCG |
| Upper_shPDCD11#1 | CCGGGCAGTCAGTTGAACAAGACAACTCGAGTTGTCTTGTTCAACTGACTGCTTTTTG |
| Bottom_shPDCD11#1  Upper_shPDCD11#2  Bottom_shPDCD11#2  Sense_siNC  Antisense_siNC  Sense_siSKP2  Antisense_siSKP2  Sense_siFBXW7  Antisense_siFBXW7  Sense_siPDCD11#1  Antisense_siPDCD11#1  Sense_siPDCD11#2  Antisense_siPDCD11#2 | AATTCAAAAAGCAGTCAGTTGAACAAGACAACTCGAGTTGTCTTGTTCAACTGACTGC  CCGGTGTTACCTCAGGACTCTATTTCTCGAGAAATAGAGTCCTGAGGTAACATTTTTG  AATTCAAAAATGTTACCTCAGGACTCTATTTCTCGAGAAATAGAGTCCTGAGGTAACA  UUCUCCGAACGUGUCACGUTT  ACGUGACACGUUCGGAGAATT  GCCUAAGCUAAAUCGAGAGAATT  UUCUCUCGAUUUAGCUUAGGCTT  ACAGGACAGUGUUUACAAATT  UUUGUAAACACUGUCCUGUTT  GCAGUCAGUUGAACAAGACAATT  UUGUCUUGUUCAACUGACUGCTT  UGUUACCUCAGGACUCUAUUUTT  AAAUAGAGUCCUGAGGUAACATT |
| sgSKP2 | TGGCAGACCTTAGACCTCACAGG |
